# Associations of Underlying Health Conditions With Anxiety and Depression Among Outpatients: Modification Effects of Suspected COVID-19 Symptoms, Health-Related and Preventive Behaviors

**DOI:** 10.3389/ijph.2021.634904

**Published:** 2021-06-23

**Authors:** Minh H. Nguyen, Thu T. M. Pham, Linh V. Pham, Dung T. Phan, Tien V. Tran, Hoang C. Nguyen, Huu C. Nguyen, Tung H. Ha, Hung K. Dao, Phuoc B. Nguyen, Manh V. Trinh, Thinh V. Do, Hung Q. Nguyen, Thao T. P. Nguyen, Nhan P. T. Nguyen, Cuong Q. Tran, Khanh V. Tran, Trang T. Duong, Tan T. Nguyen, Khue M. Pham, Lam V. Nguyen, Tam T. Vo, Binh N. Do, Nga H. Dang, Thuy T. Le, Ngoc T. Do, Hoai T. T. Nguyen, Thuy T. T. Mai, Dung T. Ha, Huong T. M. Ngo, Kien T. Nguyen, Chyi-Huey Bai, Tuyen Van Duong

**Affiliations:** ^1^ International Ph.D. Program in Medicine, College of Medicine, Taipei Medical University, Taipei, Taiwan; ^2^ Faculty of Public Health, Hai Phong University of Medicine and Pharmacy, Hai Phong, Vietnam; ^3^ School of Public Health, College of Public Health, Taipei Medical University, Taipei, Taiwan; ^4^ Department of Pulmonary and Cardiovascular Diseases, Hai Phong University of Medicine and Pharmacy Hospital, Hai Phong, Vietnam; ^5^ Director Office, Hai Phong University of Medicine and Pharmacy Hospital, Hai Phong, Vietnam; ^6^ Faculty of Nursing, Hanoi University of Business and Technology, Hanoi, Vietnam; ^7^ Nursing Office, Thien an Obstetrics and Gynecology Hospital, Hanoi, Vietnam; ^8^ Department of Infectious Diseases, Vietnam Military Medical University, Hanoi, Vietnam; ^9^ Director Office, Military Hospital 103, Hanoi, Vietnam; ^10^ Director Office, Thai Nguyen National Hospital, Thai Nguyen, Vietnam; ^11^ President Office, Thai Nguyen University of Medicine and Pharmacy, Thai Nguyen, Vietnam; ^12^ Director Office, E Hospital, Hanoi, Vietnam; ^13^ Department of Thoracic and Cardiovascular Surgery, E Hospital, Hanoi, Vietnam; ^14^ Director Office, General Hospital of Agricultural, Hanoi, Vietnam; ^15^ Director Office, Bac Ninh Obstetrics and Pediatrics Hospital, Bac Ninh, Vietnam; ^16^ Director Office, Kien An Hospital, Hai Phong, Vietnam; ^17^ Director Office, Quang Ninh General Hospital, Quang Ninh, Vietnam; ^18^ Director Office, Bai Chay Hospital, Quang Ninh, Vietnam; ^19^ Director Office, Quang Ninh Obstetrics and Pediatrics Hospital, Quang Ninh, Vietnam; ^20^ Health Management Training Institute, Hue University of Medicine and Pharmacy, Thua Thien Hue, Vietnam; ^21^ Department of Health Economics, Corvinus University of Budapest, Budapest, Hungary; ^22^ General Planning Department, Da Nang Oncology Hospital, Da Nang, Vietnam; ^23^ Director Office, Thu Duc District Health Center, Ho Chi Minh City, Vietnam; ^24^ Faculty of Health, Mekong University, Vinh Long, Vietnam; ^25^ Director Office, Hospital District 2, Ho Chi Minh City, Vietnam; ^26^ Nursing Office, Tan Phu District Hospital, Ho Chi Minh City, Vietnam; ^27^ Department of Orthopedics, Can Tho University of Medicine and Pharmacy, Can Tho, Vietnam; ^28^ Director Office, Can Tho University of Medicine and Pharmacy Hospital, Can Tho, Vietnam; ^29^ President Office, Hai Phong University of Medicine and Pharmacy, Hai Phong, Vietnam; ^30^ Aesthetic Plastic Surgery & Skin Care Center, Can Tho University of Medicine and Pharmacy Hospital, Can Tho, Vietnam; ^31^ President Office, Can Tho University of Medicine and Pharmacy, Can Tho, Vietnam; ^32^ Director Office, Trieu Phong District Health Center, Quang Tri, Vietnam; ^33^ Division of Military Science, Military Hospital 103, Hanoi, Vietnam; ^34^ Training and Direction of Healthcare Activity Center, Thai Nguyen National Hospital, Thai Nguyen, Vietnam; ^35^ Department of Quality Control, Thai Nguyen National Hospital, Thai Nguyen, Vietnam; ^36^ Faculty of Medical Laboratory Science, Da Nang University of Medical Technology and Pharmacy, Da Nang, Vietnam; ^37^ President Office, Da Nang University of Medical Technology and Pharmacy, Da Nang, Vietnam; ^38^ Nursing Office, E Hospital, Hanoi, Vietnam; ^39^ Training and Direction of Healthcare Activity Center, Kien an Hospital, Hai Phong, Vietnam; ^40^ Nursing Office, Quang Ninh General Hospital, Quang Ninh, Vietnam; ^41^ Nursing Office, Bai Chay Hospital, Quang Ninh, Vietnam; ^42^ Nursing Office, Quang Ninh Obstetric and Pediatric Hospital, Quang Ninh, Vietnam; ^43^ Department of Health Education, Faculty of Social Sciences, Behavior and Health Education, Hanoi University of Public Health, Hanoi, Vietnam; ^44^ Department of Public Health, College of Medicine, Taipei Medical University, Taipei, Taiwan; ^45^ School of Nutrition and Health Sciences, Taipei Medical University, Taipei, Taiwan

**Keywords:** COVID-19, underlying health conditions, depression, anxiety, preventive behaviors, physical activity, healthy eating, Vietnam

## Abstract

**Objectives:** We explored the association of underlying health conditions (UHC) with depression and anxiety, and examined the modification effects of suspected COVID-19 symptoms (S-COVID-19-S), health-related behaviors (HB), and preventive behaviors (PB).

**Methods:** A cross-sectional study was conducted on 8,291 outpatients aged 18–85 years, in 18 hospitals and health centers across Vietnam from 14th February to May 31, 2020. We collected the data regarding participant's characteristics, UHC, HB, PB, depression, and anxiety.

**Results:** People with UHC had higher odds of depression (OR = 2.11; *p* < 0.001) and anxiety (OR = 2.86; *p* < 0.001) than those without UHC. The odds of depression and anxiety were significantly higher for those with UHC and S-COVID-19-S (*p* < 0.001); and were significantly lower for those had UHC and interacted with “unchanged/more” physical activity (*p* < 0.001), or “unchanged/more” drinking (*p* < 0.001 for only anxiety), or “unchanged/healthier” eating (*p* < 0.001), and high PB score (*p* < 0.001), as compared to those without UHC and without S-COVID-19-S, “never/stopped/less” physical activity, drinking, “less healthy” eating, and low PB score, respectively.

**Conclusion:** S-COVID-19-S worsen psychological health in patients with UHC. Physical activity, drinking, healthier eating, and high PB score were protective factors.

## Introduction

The COVID-19 pandemic has been influencing unprecedentedly all aspects of life [1, 2]. As the figures of confirmed cases and deaths are increasing ceaselessly, it places enormous challenges on health care systems in all influenced countries [3, 4]. As effective treatment therapies and vaccines are still urgently developed [5], a variety of public health responses was promoted to contain the diffuse of COVID-19 and mitigate the negative impacts on mental health and well-being [6]. Adopting preventive behaviors and healthy lifestyles was strongly advised for the public to stay well during this difficult time [7, 8].

Compliance with preventive measures is the most recommended strategies to effectively control the spread of COVID-19 across countries [9]. Without precaution actions, the disease may spread dramatically, which may cause panic and adverse psychological outcomes in public [10]. The common protective measures, such as washing hands, wearing masks, and practicing physical distance, were highly recommended during the pandemic [11]. Therefore, engaging in preventive behaviors could help to prevent COVID-19 transmission and infection [12], which may also reduce the fear and other mental health problems [13]. Besides, maintaining active health-related behaviors plays a critical role in staying healthy during the global crisis. Recent literature has shown that following to healthy lifestyle such as doing exercise, eat healthier may help boost the immune system, reduces the risk of infection [14–16], which may further help control the unfavorable influences of the pandemic on psychological health.

People with underlying health conditions (UHC) are a vulnerable community during the COVID-19 crisis [17]. Patients with comorbidities were more likely to get serious illnesses and complications after COVID-19 infection [18]. Some recent literature showed that comorbidities were associated with an increased mortality rate among patients with COVID-19 [19–21]. These factors may increase the fear and worry of contracting COVID-19 among people with comorbid conditions. Moreover, the limitation of accessing health care systems, delay treatment, feel lack of social interaction may negatively affect their mental health [3, 22]. Therefore, it is essential to find protective factors that may help protect patients with comorbid conditions from mental illness, thereby generating timely psychological interventions.

In previous studies, we investigated the association of pre-existing health conditions with suspected COVID-19 symptoms [23], the association of lockdown measures with depression symptoms, and modifier factors of these associations [24]. However, the impact of underlying health conditions on anxiety and depression and modifier factors of these associations have not been investigated. Therefore, we conducted this study to explore the strength of the association of underlying health conditions with depression and anxiety, and examine the effect modifications of preventive behaviors, health-related behavior changes, and S-COVID-19-S on these associations among outpatients from 18 medical facilities in Vietnam.

## Methods

### Study Design and Settings

We conducted a cross-sectional study from 14th February to May 31, 2020. The study was conducted at 15 hospitals and three health centers across Vietnam, including 10 hospitals and one health center in the northern region (Military Hospital 103, E Hospital, General Hospital of Agricultural, Thai Nguyen National Hospital, Bac Ninh Obstetrics and Pediatrics Hospital, Hai Phong University of Medicine and Pharmacy Hospital, Kien An Hospital, Quang Ninh General Hospital, Bai Chay Hospital, Quang Ninh Obstetrics and Pediatrics Hospital, Kien Thuy District Health Center), one hospital and one health center in the central region (Da Nang Oncology Hospital, Trieu Phong District Health Center), four hospitals and one health center in the southern region (Thu Duc District Hospital, Hospital District 2, Tan Phu District Hospital, Can Tho University of Medicine and Pharmacy Hospital, Thu Duc District Health Center).

The study was reviewed and approved by the Institutional Ethical Review Committee of Hanoi University of Public Health, Vietnam (IRB No. 029/2020/YTCC-HD3, and IRB No. 133/2020/YTCC-HD3).

### Study Sample and Sampling

Consecutively convenience sampling was used to recruit the participants who visited the outpatient departments (OPD) at each hospital and health center. Participants recruited were those aged 18–85 years, be able to communicate using Vietnamese language, without any emergency medical conditions (e.g., stroke, respiratory failure, serious injuries). The total sample of 8,291 participants was analyzed in this study. The number of studied participants in each hospital and health center by geographic location was described in detail in a previous study [23, 24].

### Assessments and Measurements

#### Participants’ Characteristics

Participants reported their information regarding age (years), sex (male vs. female), marital status (never married vs. ever married), education (secondary school or below, high school, college/university or higher), occupation (unemployed or dependents vs. employed), ability to pay for treatments (very difficult to very easy), social status (respondents self-assessed their position in the society in terms of education, occupation, and income at three degrees of low vs. middle to high). The national lockdown measure had been applied from 1st to 22nd april across Vietnam [25, 26]. Therefore, participants who had completed the survey during this period were classified as experiencing the lockdown.

Patients self-reported body height (cm), weight (kg). Body mass index (BMI, kg/m^2^) was calculated and categorized into two groups, including normal weight (BMI < 25.0) and overweight/obese (BMI ≥ 25.0).

Patients were screened for the suspected COVID-19 symptoms (S-COVID-19-S), including common symptoms (fever, cough, dyspnea), and less common symptoms (myalgia, fatigue, sputum production, confusion, headache, sore throat, rhinorrhea, chest pain, hemoptysis, diarrhea, and nausea/vomiting) [27]. We classified participants as having S-COVID-19-S if they reported any of these symptoms.

### Underlying Health Conditions

The underlying health problems were assessed using the Charlson Comorbidity Index items [28]. Participants were asked whether they had one of the following medical conditions, including myocardial infarction, congestive heart failure, peripheral disease, cerebrovascular disease, chronic pulmonary disease, diabetes without complications, taking anticoagulants (for cardiovascular disease, thrombosis), hemiplegia, diabetes with complications, moderate or severe renal disease, tumor without metastasis, moderate or severe liver disease, solid metastatic tumor, HIV/AIDS. We categorized underlying health conditions (UHC) into two groups: with UHC vs. without UHC. Patients were classified as having UHC if they had at least one of these above diseases.

### Health Literacy

The short form of the health literacy questionnaire (HLS-SF12) was used to measure health literacy (HL). This questionnaire has been validated and used in Asia [29], including in Vietnam [30] with satisfactory reproductivity and validity. The HLS-SF12 consists of 12 items assessing participant’s perceived difficulty on 4-point Likert responses from 1 (very difficult) to 4 (very easy). We transformed the HL score into a standardized index ranging from 0 to 50, with higher scores indicating better health literacy, using the formula:

Index=(mean−1)×(50/3) (1)

In which, *Index* is the standardized HL index score, *mean* is the average score of 12 questionnaire items for each participant, one is the lowest value of the mean, three is the range of the mean, and 50 is the highest value of the index. In the current study, the Cronbach’s α score of the HLS-SF12 was 0.94.

### Health-Related Behavior Changes

Patients provided their information about current lifestyle behaviors in comparison to before the epidemic. Smoking, drinking, and physical activity were assessed on five responses: never, stopped, less, unchanged, and more. Eating behavior also was reported on three responses scale from less healthy, unchanged, and healthier. Based on the WHO’s guideline [9], people were recommended to keep their positive lifestyles (e.g., physical exercise, balanced diet) unchanged or better to stay healthy during the COVID-19 crisis. Thus, we classified the “unchanged or healthier” eating and “unchanged or more” physical activity as healthy behavior changes and “unchanged or more” smoking or drinking as unhealthy behavior changes. Patient’s responses were classified into two groups: “never/stopped/less” vs. “unchanged or more” for smoking, drinking, and physical activity, “less healthy” vs. “unchanged or healthier” for the eating behavior.

### Preventive Behaviors

Preventive behaviors were evaluated through three items based on the COVID-19 prevention guidelines of the world health organization [9], including 1) washing hands with soap or alcohol-based liquid, 2) wearing masks when getting outside, 3) adherence to at least 2 m physical distance rule. Participants reported how often they followed those preventive behaviors during the pandemic on a 5-point Likert scale ranging from 0 (Never) to 4 (Always). The total score ranges from 0 to 12, with a higher score indicates higher compliance with preventive behaviors. We categorized PB into two categories based on the median value of 9 [31], including low PB score (< 9) and high PB score (≥ 9). The Cronbach’s Alpha score of this questionnaire in this study was 0.81.

### Psychological Health

Depression was assessed using the Patient health questionnaire (PHQ-9), which was widely used in Vietnam [24]. The PHQ-9 has nine items regarding the frequency of given symptoms that participants had encountered over the past two weeks, and the score of each item is given on a 4-point scale from 0 (not at all) to 3 (almost every day). The total PHQ score ranges from 0 to 27, and scores ≥10 were classified as having depression symptoms [32].

Anxiety was assessed using the Generalized Anxiety Disorder (GAD-7) [33], which was validated and used in Vietnam [34]. The GAD-7 comprises seven items about how often they have been encountered seven symptoms over the last two weeks on the 4-point Likert scale ranging from 0 (not at all) to 3 (nearly every day). The total GAD score ranges from 0 to 21, and scores ≥8 were classified as having anxiety disorders [35]. The Cronbach’s Alpha index for the PHQ-9 and the GAD-17 in this study were 0.90 and 0.94, respectively.

### Data Collection Procedure

Research assistants (physicians, nurses, and medical students) received a 4 h training session for data collection which conducted by researchers in each medical facility. The infection control training (e.g., mask-wearing, handwashing, distancing) were also provided using guidelines of the Centers for disease Control and Prevention (CDC) [36], and World Health Organization [37].

Research assistants (e.g., physicians, nurses, medical students) invited and asked for voluntary participation of OPD visitors. All eligible participants have signed the informed consent form before conducting the survey. We collected data through a self-administered questionnaire. In the initial period, only printed self-administered questionnaires were used for data collection. However, at the peak of the pandemic, patients can choose to complete the survey by filling in the printed questionnaire or scanning the printed QR codes and completed the survey using their smartphone while waiting for the doctor’s visit. During the questionnaire administration, research assistants provided guidance and support if it’s necessary. Each survey took about 20–30 min to complete. Finally, the data was cleaned and analyzed by researchers confidentially.

### Statistical Analysis

The sample of 4,348 participants was used for analysis of preventive behaviors and anxiety as data of these variables were collected in the peak stage of the pandemic. A sample of 8,291 participants was used for analysis of all other variables which investigated in two stages of the pandemic. First, the Chi-square test was performed to examine the distribution of depression and anxiety among different studied variables. Secondly, the associations of underlying health conditions, S-COVID-19-S, health-related behavior changes and PB with depression and anxiety were tested separately in different models using the simple and adjusted logistic regression models. To reduce the impact of residual confounders, age, gender, and factors associated with outcomes at *p* < 0.20 in the bivariate models were chosen to adjust in multivariate models. To eliminate multicollinearity, the Spearman correlation was utilized to examine correlations between potential confounders. If the correlation between two confounders at rho ≥0.30, one representative confounder was adjusted in final models. The results were presented in Supplementary Tables S1–S3 in the Supplementary File. Finally, the interaction analyses were performed (using the interaction terms) to examine the effect modification of S-COVID-19-S, health-related behavior changes, and preventive behaviors on the associations of UHC with anxiety and depression. Model 1 (unadjusted model) was performed with three terms, including X1, X2, and X1*X2 (where X1 is the main effect of UHC, and X2 is the main effect of modifying factors. Model 2 (adjusted model) was conducted with three terms in model 1 and adjusted for potential confounders. The interaction models were presented with the odds ratio and the 95% confidence interval of three terms (X1, X2, and the interaction of X1*X2). The *p*-value < 0.05 was considered significance. We analyzed data using the IBM SPSS Version 20.0 (IBM Corp, Armonk, NY, United States).

## Results

### Demographic Characteristics

The mean age, HL of the population were 43.6 ± 16.9, 28.1 ± 9.4, respectively. The prevalence of underlying health conditions (UHC), depression, and anxiety disorders were 22.3% (1867/8,291), 12.5% (1,033/8,291), and 16.7% (726/4,348), respectively. Of 8,291 participants, 41% were men, 79.9% ever married, 45.5% had college/university or higher degree, 92.5% had a job, 45.9 reported felt very or fairly easy to pay for treatments, 28.7% participated in surveying during the lockdown period, 11.7% were overweight or obese, and 37.7% had S-COVID-19-S. In the pandemic, the proportions of patients who responded “unchanged/healthier” eating behavior, “unchanged/more” smoking, drinking, and physical activity were 94.9, 9.0, 14.9, and 66.1%, respectively (Table 1).

**TABLE 1:** Characteristics of study participants, Vietnam, 2020.

Variables		Depression (*n* = 8,291)			Anxiety (*n* = 4,348)		
	Total (*n* = 8,291)	PHQ < 10 (*n* = 7,258)	PHQ ≥ 10 (*n* = 1,033)	p*	GAD < 8 (*n* = 3,621)	GAD ≥ 8 (*n* = 726)	p*
	n (%)	n (%)	n (%)		n (%)	n (%)	
Age, year (mean ± SD)	43.6 ± 16.9	—	—	<0.001	—	—	<0.001
18–39	3,955 (47.7)	3,688 (50.8)	267 (25.8)	—	2014 (55.6)	154 (21.2)	—
40–59	2,473 (29.8)	2,220 (30.6)	253 (24.5)	—	1,075 (29.7)	168 (23.1)	—
≥60	1863 (22.5)	1,350 (18.6)	513 (49.7)	—	532 (14.7)	404 (55.6)	—
Gender	—	—	—	0.906	—	—	0.182
Women	4,890 (59.0)	4,279 (59.0)	611 (59.1)	—	2,260 (62.4)	434 (59.8)	—
Men	3,401 (41.0)	2,979 (41.0)	422 (40.9)	—	1,361 (37.6)	292 (40.2)	—
Marital status	—	—	—	<0.001	—	—	<0.001
Never married	1,635 (19.7)	1,496 (20.7)	139 (13.5)	—	709 (19.7)	63 (8.7)	—
Ever married	6,628 (79.9)	5,734 (79.3)	894 (86.5)	—	2,896 (80.3)	663 (91.3)	—
Education attainment	—	—	—	0.024	—	—	<0.001
Secondary school or below	2,223 (26.8)	1911 (26.4)	312 (30.2)	—	786 (21.7)	221 (30.4)	—
High school	2,277 (27.5)	1995 (27.5)	282 (27.3)	—	986 (27.3)	209 (28.8)	—
College/university or higher	3,776 (45.5)	3,337 (46.1)	439 (42.5)	—	1843 (51.0)	296 (40.8)	—
Occupation	—	—	—	<0.001	—	—	<0.001
Unemployed/ Dependents	623 (7.5)	467 (6.4)	156 (15.1)	—	349 (9.6)	125 (17.2)	—
Employed	7,668 (92.5)	6,791 (93.6)	877 (84.9)	—	3,272 (90.4)	601 (82.8)	—
Ability to pay for treatments	—	—	—	<0.001	—	—	<0.001
Very or fairly difficult	4,475 (54.0)	3,710 (51.2)	765 (74.1)	—	2,115 (58.6)	596 (82.1)	—
Very or fairly easy	3,805 (45.9)	3,537 (48.8)	268 (25.9)	—	1,496 (41.4)	130 (17.9)	—
Social status	—	—	—	<0.001	—	—	<0.001
Low	1,403 (16.9)	1,187 (16.4)	216 (20.9)	—	803 (22.2)	118 (16.3)	—
Middle or high	6,879 (83.0)	6,062 (83.6)	817 (79.1)	—	2,810 (77.8)	608 (83.7)	—
Lockdown measure	—	—	—	<0.001	—	—	<0.001
No	5,915 (71.3)	5,428 (74.8)	487 (47.1)	—	1789 (49.4)	183 (25.2)	
Yes	2,376 (28.7)	1830 (25.2)	546 (52.9)	—	1832 (50.6)	543 (74.8)	
BMI, kg/m^2^	—	—	—	0.999	—	—	0.616
Normal weight (BMI <25.0)	7,301 (88.1)	6,394 (88.2)	907 (88.2)	—	3,163 (87.5)	627 (86.8)	—
Overweight/obese (BMI ≥25.0)	974 (11.7)	853 (11.8)	121 (11.8)	—	451 (12.5)	95 (13.2)	—
Underlying health conditions (UHC)	—	—	—	<0.001	—	—	<0.001
Non-UHC	6,434 (77.7)	5,893 (81.3)	541 (52.4)		2,805 (77.5)	298 (41.0)	—
UHC	1848 (22.3)	1,357 (18.7)	491 (47.6)		816 (22.5)	428 (59.0)	—
S-COVID-19-S^a^	—	—	—	<0.001	—	—	<0.001
No	5,162 (62.3)	4,827 (66.5)	335 (32.4)	—	2,375 (65.6)	219 (30.2)	—
Yes	3,129 (37.7)	2,431 (33.5)	698 (67.6)	—	1,246 (34.4)	507 (69.8)	—
Smoking tobacco^b^	—	—	—	0.867	—	—	0.643
Never, stopped, or smoke less	7,541 (91.0)	6,600 (90.9)	941 (91.1)	—	3,323 (91.8)	670 (92.3)	—
Unchanged or smoke more	750 (9.0)	658 (9.1)	92 (8.9)	—	298 (8.2)	56 (7.7)	—
Drinking alcohol^b^	—	—	—	0.680	—	—	0.001
Never, stopped, or drink less	7,044 (85.0)	6,178 (85.1)	866 (84.7)	—	3,372 (93.2)	642 (89.7)	—
Unchanged or drink more	1,235 (14.9)	1,078 (14.9)	157 (15.3)	—	247 (6.8)	74 (10.3)	—
Physical activity^b^	—	—	—	<0.001	—	—	<0.001
Never, stopped, or exercise less	2,778 (33.5)	2,190 (30.3)	588 (57.1)	—	1960 (54.1)	555 (76.4)	—
Unchanged or exercise more	5,480 (66.1)	5,038 (69.7)	442 (42.9)	—	1,661 (45.9)	171 (23.6)	—
Eating behavior^b^	—	—	—	<0.001	—	—	<0.001
Less healthy	383 (4.6)	193 (2.7)	190 (18.4)	—	144 (4.0)	181 (24.9)	—
Unchanged or healthier	7,868 (94.9)	7,026 (97.3)	842 (81.6)	—	3,456 (96.0)	545 (75.1)	—
PB score, median (IQR)^c^	9.0 (8.0—10.0)	—	—	<0.001	—	—	<0.001
PB score <9.0	1744 (40.1)	1,241 (34.4)	503 (68.1)	—	1,225 (33.8)	519 (71.5)	—
PB score ≥9.0	2,604 (59.9)	2,368 (65.6)	236 (31.9)	—	2,396 (66.2)	207 (28.5)	—
HL score (mean ± SD)	28.1 ± 9.4	—	—	—	—	—	—

PHQ, patient health questionnaire; GAD, Generalized Anxiety Disorder; SD, standard deviation; UHC, underlying health conditions; S-COVID-19-S, suspected corona virus disease-2019 symptoms; BMI, body mass index; PB, preventive behavior; IQR, interquartile range; HL, health literacy.

*Results of the Chi-square test.

a The suspected COVID-19 symptoms including common symptom (fever, cough, dyspnea), less common symptom (myalgia, fatigue, sputum production, confusion, headache, sore throat, rhinorrhea, chest pain, hemoptysis, diarrhea, and nausea/vomiting).

b People were asked whether their health-related behaviors are getting worse, better, or unchanged during COVID-19 pandemic as compared to those before the pandemic.

c Preventive behaviors was analyzed on the sample of 4,348 participants with two outcomes (depression and anxiety).

### Underlying Health Conditions and Mental Health Outcomes

Table 2 presents the association of underlying health conditions with depression and anxiety symptoms. After adjusted for confounders, multiple logistic regression models showed that participants with UHC had higher odds of experiencing depression (OR, 2.11; 95% CI, 1.81–2.46; *p* < 0.001) and anxiety symptoms (OR, 2.86; 95% CI, 2.37–3.46; *p* < 0.001), as compared to those without comorbidity (Table 2). The odds of having depression and anxiety symptoms were significantly lower in patients who had a high PB score (OR, 0.36; 95% CI, 0.30–0.44; *p* < 0.001) and (OR, 0.28; 95% CI, 0.23–0.34; *p* < 0.001), who had physical activity at “unchanged or more” level (OR, 0.53; 95% CI, 0.45–0.61; *p* < 0.001) and (OR, 0.48; 95% CI, 0.39–0.59; *p* < 0.001), who ate “unchanged or healthier” (OR, 0.22; 95% CI, 0.17–0.28; *p* < 0.001) and (OR, 0.20; 95% CI, 0.15–0.26; *p* < 0.001), respectively, as compared to counterparts. The odds of depression and anxiety symptoms were significantly higher in patients with S-COVID-19-S (OR, 2.84; 95% CI, 2.45–3.29); *p* < 0.001) and (OR, 2.92; 95% CI, 2.42–3.52; *p* < 0.001), respectively. Besides, the odds of suffering from anxiety symptoms were significantly larger in patients who drunk at “unchanged or more” level (OR, 1.99; 95% CI, 1.45–2.72; *p* < 0.001).

**TABLE 2:** Associations of underlying health conditions, suspected COVID-19 symptoms, health-related behavior changes, and preventive behaviors with depression, and anxiety using logistic regression models, Vietnam, 2020.

Variable	Depression (PHQ ≥ 10)				Anxiety (GAD ≥ 8)^a^			
	Model 1		Model 2		Model 1		Model 2	
	OR (95% CI)	p	OR (95% CI)	p	OR (95% CI)	p	OR (95% CI)	p
Underlying health conditions								
Non-UHC	1.00	—	1.00	—	1.00	—	1.00	—
UHC	3.94 (3.44, 4.51)	<0.001	2.11 (1.81, 2.46)	<0.001	4.94 (4.18, 5.83)	<0.001	2.86 (2.37, 3.46)	<0.001
S-COVID-19-S								
No	1.00	—	1.00	—	1.00	—	1.00	—
Yes	4.14 (3.60, 4.75)	<0.001	2.84 (2.45, 3.29)	<0.001	4.41 (3.71, 5.24)	<0.001	2.92 (2.42, 3.52)	<0.001
Smoking tobacco								
Never, stopped, or smoke less	1.00	—	1.00	—	1.00	—	1.00	—
Unchanged or smoke more	0.98 (0.78, 1.23)	0.867	1.08 (0.84, 1.38)	0.550	0.93 (0.69, 1.25)	0.643	1.02 (0.73, 1.42)	0.895
Drinking alcohol								
Never, stopped, or drink less	1.00	—	1.00	—	1.00	—	1.00	—
Unchanged or drink more	1.04 (0.86, 1.25)	0.680	1.19 (0.98, 1.45)	0.081	1.57 (1.19, 2.07)	0.001	1.99 (1.45, 2.72)	<0.001
Physical activity								
Never, stopped, or exercise less	1.00	—	1.00	—	1.00	—	1.00	—
Unchanged or exercise more	0.33 (0.28, 0.37)	<0.001	0.53 (0.45, 0.61)	<0.001	0.36 (0.30, 0.44)	<0.001	0.48 (0.39, 0.59)	<0.001
Eating behavior								
Less healthy	1.00	—	1.00	—	1.00	—	1.00	—
Unchanged or healthier	0.12 (0.09, 0.15)	<0.001	0.22 (0.17, 0.28)	<0.001	0.12 (0.10, 0.16)	<0.001	0.20 (0.15, 0.26)	<0.001
Preventive behaviors^b^								
Low PB score (<9.0)	1.00	—	1.00	—	1.00	—	1.00	—
High PB score (≥9.0)	0.25 (0.21, 0.29)	<0.001	0.36 (0.30, 0.44)	<0.001	0.20 (0.17, 0.24)	<0.001	0.28 (0.23, 0.34)	<0.001

PHQ, patient health questionnaire; GAD, Generalized Anxiety Disorder; UHC, underlying health conditions; S-COVID-19-S, suspected corona virus disease-2019 symptoms; PB, preventive behaviors; OR, odd ratio; CI, confidence interval.

Model 1 Association of UHC, S-COVID-19-S, health-related behavior changes, PB with depression, and anxiety.

Model 2 The model 1 was adjusted for age (for both outcomes), gender (for both outcomes), occupation (for both outcomes), ability to pay for treatments (for depression only), lockdown measure (for both outcomes), and social status (for both outcomes) (Supplementary Tables S1–S3 in Supplementary File).

Independent variables were analyzed separately in different models. Full models for UHC, S-COVID-19-S, smoking, drinking alcohol, physical activity, eating behavior, and PB are presented in Supplementary Tables S4–S10 in Supplementary File, respectively.

a Anxiety was analyzed on the sample of 4,348 participants.

b Participants was asked how often they followed preventive behaviors, including washing hands, wearing masks, and physical distancing, during the pandemic. PB was analyzed on the sample of 4,348 participants with two outcomes (depression and anxiety).

### Effect Modifications of S-COVID-19-S, Health-Related and Preventive Behaviors

In the interaction analysis models, we found that as compared to participants without UHC and without S-COVID-19-S, those without UHC and with S-COVID-19-S had 1.92 times higher likelihood of having depressive symptoms (OR, 1.92; 95% CI, 1.59–2.31; *p* < 0.001), and 1.50 times higher likelihood of anxiety symptoms (OR, 1.50; 95% CI, 1.15–1.96; *p* = 0.003); while those with both UHC and S-COVID-19-S had 2.62 times higher depressive symptoms likelihood (OR, 2.62; 95% CI, 1.81–3.79; *p* < 0.001), and 3.12 times higher anxiety symptoms likelihood (OR, 3.12; 95% CI, 1.92–5.07; *p* < 0.001; Table 3).

**TABLE 3:** Interactions of underlying health conditions with suspected COVID-19 symptoms on depression, and anxiety, Vietnam, 2020.

Interaction	Depression (PHQ ≥ 10)				Anxiety (GAD ≥ 8)^a^			
	Model 1		Model 2		Model 1		Model 2	
	OR (95% CI)	*p*	OR (95% CI)	*p*	OR (95% CI)	*p*	OR (95% CI)	*p*
Non-UHC × without S-COVID-19-S	1.00	—	1.00	—	1.00	—	1.00	—
UHC × without S-COVID-19-S	1.34 (0.98, 1.81)	0.063	0.84 (0.61, 1.16)	0.293	1.76 (1.19, 2.61)	0.004	0.92 (0.61, 1.39)	0.685
Non-UHC × with S-COVID-19-S	2.34 (1.96, 2.79)	<0.001	1.92 (1.59, 2.31)	<0.001	2.00 (1.56, 2.58)	<0.001	1.50 (1.15, 1.96)	0.003
UHC × with S-COVID-19-S	2.77 (1.94, 3.94)	<0.001	2.62 (1.81, 3.79)	<0.001	2.18 (1.38, 3.45)	0.001	3.12 (1.92, 5.07)	<0.001

PHQ, patient health questionnaire; GAD, Generalized Anxiety Disorder; OR, odd ratio; CI, confidence interval; UHC, underlying health conditions; S-COVID-19-S, suspected corona virus disease-2019 symptoms.

Model 1 Simple logistic regression to test the main effect of UHC, the main effect of S-COVID-19-S, and the interaction of UHC with S-COVID-19-S on anxiety and depression**.**

Model 2 The model 1 was adjusted for age (for both outcomes), gender (for both outcomes), occupation (for both outcomes), ability to pay for treatments (for depression only), lockdown measure (for both outcomes), and social status (for both outcomes) (Supplementary Tables S1–S3 in Supplementary File). The full model is presented in Supplementary Table S11 in Supplementary File.

a Anxiety was analyzed on the sample of 4,348 participants.

In comparison to patients without UHC and drinking at “never/stopped/less” level, those without UHC and drinking at “unchanged or more” level had 3.26 times higher likelihood of having anxiety symptoms (OR, 3.26; 95% CI, 2.28–4.65; *p* < 0.001); those with UHC and drinking at “never/stopped/less” level had 3.31 times higher likelihood of developing anxiety disorders (OR, 3.31; 95% CI, 2.71–4.05; *p* < 0.001); while participants with UHC and drinking at “unchanged or more” level had 66% lower likelihood of experiencing anxiety symptoms (OR, 0.34; 95% CI, 0.16–0.69; *p* = 0.003; Table 4).

**TABLE 4:** Interactions of underlying health conditions with health-related behavior changes on depression, and anxiety, Vietnam, 2020.

Interaction	Depression (PHQ ≥10)				Anxiety (GAD ≥8)^a^			
	Model 1		Model 2		Model 1		Model 2	
	OR (95% CI)	*p*	OR (95% CI)	*p*	OR (95% CI)	*p*	OR (95% CI)	*p*
Interaction of comorbidity with drinking alcohol								
Non-UHC × never, stopped, or drink less	—	—	—	—	1.00	—	1.00	—
UHC × never, stopped, or drink less	—	—	—	—	5.71 (4.77, 6.83)	<0.001	3.31 (2.71, 4.05)	<0.001
Non-UHC × unchanged or drink more	—	—	—	—	3.05 (2.21, 4.21)	<0.001	3.26 (2.28, 4.65)	<0.001
UHC × unchanged or drink more	—	—	—	—	0.25 (0.12, 0.48)	<0.001	0.34 (0.16, 0.69)	0.003
Interaction of comorbidity with physical activity								
Non-UHC × never, stopped, or exercise less	1.00	—	1.00	—	1.00	—	1.00	—
UHC × never, stopped, or exercise less	5.26 (4.34, 6.39)	<0.001	3.03 (2.45, 3.74)	<0.001	6.08 (4.96, 7.46)	<0.001	3.46 (2.76, 4.35)	<0.001
Non-UHC × unchanged or exercise more	0.58 (0.48, 0.69)	<0.001	0.77 (0.64, 0.94)	0.010	0.68 (0.53, 0.86)	0.002	0.75 (0.58, 0.97)	0.027
UHC × unchanged or exercise more	0.36 (0.27, 0.48)	<0.001	0.42 (0.30, 0.56)	<0.001	0.34 (0.23, 0.50)	<0.001	0.42 (0.28, 0.64)	<0.001
Interaction of comorbidity with eating behavior								
Non-UHC × less healthy	1.00	—	1.00	—	1.00	—	1.00	—
UHC × less healthy	10.92 (6.79, 17.56)	<0.001	4.27 (2.57, 7.08)	<0.001	16.97 (9.78, 29.45)	<0.001	6.27 (3.46, 11.35)	<0.001
Non-UHC × unchanged or healthier	0.30 (0.21, 0.42)	<0.001	0.36 (0.25, 0.52)	<0.001	0.35 (0.23, 0.53)	<0.001	0.36 (0.23, 0.56)	<0.001
UHC × unchanged or healthier	0.28 (0.17, 0.45)	<0.001	0.42 (0.25, 0.72)	0.001	0.21 (0.12, 0.38)	<0.001	0.37 (0.20, 0.68)	0.002

PHQ, patient health questionnaire; GAD, Generalized Anxiety Disorder; OR, odd ratio; CI, confidence interval; UHC, underlying health conditions.

Model 1 Simple logistic regression to test the main effect of UHC, the main effect of health-related behavior changes, and the interaction of UHC with health-related behavior changes on anxiety and depression.

Model 2 The model 1 was adjusted for age (for both outcomes), gender (for both outcomes), occupation (for both outcomes), ability to pay for treatments (for depression only), lockdown measure (for both outcomes), and social status (for both outcomes) (Supplementary Tables S1–S3 in Supplementary File).

Full models for drinking alcohol, physical activity, and eating behaviors are presented in Supplementary Tables S12–S14 in Supplementary File.

a Anxiety was analyzed on the sample of 4,348 participants.

In comparison to patients without UHC and had physical activity at “never/stopped/less” level, those without UHC and did physical activity at “unchanged or more” level had a 23% lower likelihood of depressive symptoms (OR, 0.77; 95% CI, 0.64–0.94; *p* = 0.01) and a 25% lower anxiety symptoms likelihood (OR, 0.75; 95% CI, 0.58–0.97; *p* = 0.027); those with UHC and had physical activity at “never/stopped/less” level had 3.03 times higher likelihood of depressive symptoms (OR, 3.03; 95% CI, 2.45–3.74; *p* < 0.001) and 3.46 times higher anxiety symptoms likelihood (OR, 3.46; 95% CI, 2.76–4.35; *p* < 0.001); while patients with UHC and had physical activity at “unchanged or more” level had 58% lower depressive symptoms likelihood (OR, 0.42; 95% CI, 0.30–0.56; *p* < 0.001) and 58% lower anxiety symptoms likelihood (OR, 0.42; 95%CI, 0.28–0.64; *p* < 0.001; Table 4).

In comparison to people without UHC and ate “less healthy”; those without UHC and ate “unchanged or healthier” had a 64% lower likelihood of developing depressive symptoms (OR, 0.36; 95% CI, 0.25–0.52; *p* < 0.001), and a 64% lower likelihood of having anxiety symptoms (OR, 0.36; 95% CI, 0.23–0.56; *p* < 0.001); those with UHC and ate “less healthy” had 4.27 times higher likelihood of depressive symptoms (OR, 4.27; 95% CI, 2.57–7.08; *p* < 0.001) and 6.27 times higher likelihood of anxiety disorders (OR, 6.27; 95% CI, 3.46–11.35; *p* < 0.001); while patients with UHC and ate “unchanged or healthier” had 58% lower likelihood of depressive symptoms (OR, 0.42; 95% CI, 0.25–0.72; *p* = 0.001), and a 63% lower anxiety symptoms likelihood (OR, 0.37; 95% CI, 0.20–0.68; *p* = 0.002; Table 4).

In comparison to participants without UHC and with a low PB score; those without UHC and with a high PB score had a 43% lower likelihood of depressive symptoms (OR, 0.57; 95% CI, 0.44–0.73; *p* < 0.001) and a 45% lower anxiety symptoms likelihood (OR, 0.55; 95% CI, 0.43–0.71; *p* < 0.001); those with UHC and with a low PB score had 5.32 times higher likelihood of depressive symptoms (OR, 5.32; 95% CI, 4.11–6.89; *p* < 0.001) and 6.24 times lower anxiety symptoms likelihood (OR, 6.24; 95% CI, 4.80–8.11; *p* < 0.001); while participants with UHC and with a high PB score had 69% lower depressive symptoms likelihood (OR, 0.31; 95% CI, 0.21–0.45; *p* < 0.001) and 82% lower anxiety symptoms likelihood (OR, 0.18; 95% CI, 0.12–0.27; *p* < 0.001; Table 5).

**TABLE 5:** Interactions of underlying health conditions with preventive behaviors on depression, and anxiety, Vietnam, 2020^a^.

Interaction	Depression (PHQ ≥ 10)				Anxiety (GAD ≥ 8)			
	Model 1		Model 2		Model 1		Model 2	
	OR (95% CI)	*p*	OR (95% CI)	*p*	OR (95% CI)	*p*	OR (95% CI)	*p*
Non-UHC × PB score <9.0	1.00	—	1.00	—	1.00	—	1.00	—
UHC × PB score <9.0	9.83 (7.75, 12.46)	<0.001	5.32 (4.11, 6.89)	<0.001	11.46 (9.02, 14.58)	<0.001	6.24 (4.80, 8.11)	<0.001
Non-UHC × PB score ≥9.0	0.49 (0.38, 0.62)	<0.001	0.57 (0.44, 0.73)	<0.001	0.48 (0.37, 0.61)	<0.001	0.55 (0.43, 0.71)	<0.001
UHC × PB score ≥9.0	0.21 (0.15, 0.30)	<0.001	0.31 (0.21, 0.45)	<0.001	0.14 (0.09, 0.19)	<0.001	0.18 (0.12, 0.27)	<0.001

PHQ, patient health questionnaire; GAD, Generalized Anxiety Disorder; OR, odd ratio; CI, confidence interval; UHC, underlying health conditions; PB, preventive behaviors.

Model 1 Simple logistic regression to test the main effect of UHC, the main effect of preventive behaviors, and the interaction of UHC with preventive behaviors on anxiety and depression.

Model 2 The model 1 was adjusted for age (for both outcomes), gender (for both outcomes), occupation (for both outcomes), ability to pay for treatments (for depression only), lockdown measure (for both outcomes), and social status (for both outcomes) (Supplementary Tables S1–S3 in Supplementary File). The full model is presented in Supplementary Table S15 in Supplementary File.

a PB score and Anxiety were analyzed on the sample of 4,348 participants.

## Discussion

The present study showed that individuals with underlying health conditions are more likely to have depression and anxiety symptoms. The results were similar to results from previous studies [38, 39]. This association could be explained that underlying diseases may directly develop mental illnesses [40]. In addition, in the pandemic, people with comorbidities are vulnerable to severity and mortality after COVID-19 infection [19–21, 41]. They also were recommended to stay at home as much as possible to reduce the chance of infection. Therefore, the feeling of isolation and the fear of catching COVID-19 may harm their mental health during the pandemic [42].

In the interaction analysis, we found that people who had both underlying health conditions and suspected COVID-19 symptoms had higher odds of depression and anxiety symptoms than those without. Previous studies found that individuals with suspected COVID-19 symptoms, and those who had chronic conditions were positively associated with suffering from psychological illnesses during the pandemic [39, 43, 44]. Thus, it is understandable that individuals suffering from double burden may more likely experience adverse psychological consequences in the pandemic.

We also found that people with comorbidities who highly adhered to preventive behaviors had lower odds of experiencing depressive and anxiety symptoms than those who less adhered to. Previous research in Turkey found that compliance with preventive behaviors was associated with better psychological outcomes in the pandemic [13]. The benefits of handwashing and mask-wearing on reducing the harmful impact of the pandemic on psychological health also was discussed in previous studies [39, 45]. Preventive measures such as washing hands, wearing a mask, and physical distancing may help reduce the risk of coronavirus transmission, especially for people with underlying medical conditions who often access healthcare services for treatments. Although physical distancing reduces social interaction [42, 46], which may negatively influence mental health, for high-risk populations like people with underlying health conditions, preventing coronavirus infection is more critical [19]. Generally, preventive behaviors may help patients with comorbid conditions decrease the fear and worry of serious infections, which may improve their mental health. Therefore, governments should encourage the public to adhere to preventive behaviors to control the disease and reduce adverse psychological effects, particularly in low and middle-income countries, when the proportion of handwashing was still low [47].

The current study shows that keeping positive behaviors, including physical activity and healthy eating, unchanged or better could help reduce the negative impacts of underlying health conditions on psychological health during the pandemic. Previous studies showed that individuals doing exercise routinely were associated with better health outcomes across various physical diseases [48, 49], which may help ameliorate psychological health. Physical activities also were recommended as an effective therapy for depression [50]. Better nutrition also was also associated with a declined occurrence of functional disability and mortality rate [51]. Besides, the effect of higher quality diets on reducing the risk of being depressed has been discussed in previous studies [52, 53]. Notably, in the COVID-19 epidemic, staying physical activities and better diets have been suggested to enhance the immune system and alleviate the harmful effects of being isolated [54–56]. A recent study has shown that healthy dietary intake help reduces the occurrence of depression in the outpatient sample during the lockdown period [24]. Moreover, other studies have indicated that people with higher health literacy are more likely to maintain physical exercise and healthy eating behaviors [57, 58]. Therefore, timely health education strategies with the participation of the government and health professionals should be promoted to reduce sedentary lifestyle and improve meal quality for high-risk groups during and after the COVID-19 pandemic.

In multiple logistic regression, our findings found that patients who kept drinking habits unchanged or more had a higher likelihood of developing anxiety symptoms during a pandemic, which is consistent with findings from previous studies [59]. However, in interaction analyses, “unchanged or more” drinking could help people with comorbidities mitigate anxiety symptoms. Previous studies found that individuals with greater stress or fear of COVID-19 were more likely to drink more alcohol as a coping method during the pandemic [60–62]. Drinking may immediately help reduce tension and worry in uncomfortable circumstances [63, 64]. However, in the long term, alcohol abuse may have a harmful effect on both physical and mental health [65, 66], particularly people with underlying medical conditions. Therefore, other effective coping strategies should be proposed to improve mental health and reduce coping-motivated drinking for high-risk groups during the pandemic.

This study has some limitations. First, as the investigation was carried out at the time of the COVID-19 crisis, both research assistants and studied patients were at great risk of COVID-19 infection. Thus, all medical facilities, researchers were required to strictly follow the national guidelines of self-prevention throughout the data-collecting process to avoid the spread of COVID-19. During the pandemic, people exposed to the risks were immediately quarantined, and people infected were immediately isolated in the authorized institutions. Therefore, we could not approach those people to investigate their real experiences. In addition, there was no new confirmed case of COVID-19 reported in studied hospitals during the period of the study [67]. Secondly, in this study, we investigated the drinking behavior changes but not alcohol abuse or drinking frequency which may not provide the in-depth analysis of the association. In addition, the cultural aspect was also not investigated. Future studies should take these aspects into account of measurements. Thirdly, the socially desirable responses regarding healthy behavior in the context of a healthcare facility may cause the reporting bias. To minimize this bias, we encouraged participants to provide the most accurate information and ensured confidentiality. In this study, we used two types of the administered questionnaire for data collection, including the printed version and the online version. Therefore, we created a new dummy variable for the type of the administered questionnaire (online version vs. printed version) and adjusted it with other confounders in the final models. There is no significant difference in the results of the final models. These results were presented in Supplementary Tables S16–S19 in the Supplementary File. Finally, a causal relationship cannot be drawn from a cross-sectional study. However, with relatively large sample size, our findings might be helpful to raise an important phenomenon and provide evidence for developing appropriate interventions to minimize the psychological effects of individuals, especially those with comorbidity during the COVID-19 pandemic. Future studies need to perform with a longitudinal design to confirm the current findings.

### Conclusion

In the current study, we found that people with underlying health conditions are more vulnerable to depression and anxiety symptoms. Having S-COVID-19-S made these patients more depressed and anxious. However, positive changes in lifestyles (e.g., doing more physical exercising, healthier eating) and adherence to protective measures showed benefits for mental health in those with underlying diseases. Unexpectedly, the constant or increase drinking behavior was found as a coping behavior to protect patients against psychological illnesses during the pandemic. Our findings could be helpful for governments and health organizations to develop appropriate strategies to alleviate the harmful impact of the pandemic on mental health, especially in patients with underlying health conditions.

## Data Availability

The raw data supporting the conclusions of this article will be made available on reasonable request to the corresponding authors.

## References

[1] VanderWeele TJ. Challenges Estimating Total Lives Lost in COVID-19 Decisions. Jama (2020). 324:445–6. 10.1001/jama.2020.12187 32639547

[2] Watkins J Wulaningsih W. Three Further Ways that the COVID-19 Pandemic Will Affect Health Outcomes. Int J Public Health (2020). 65:519–20. 10.1007/s00038-020-01383-6 32372270 PMC7199867

[3] Armocida B Formenti B Ussai S Palestra F Missoni E. The Italian Health System and the COVID-19 challenge. The Lancet Public Health (2020). 5:E253. 10.1016/S2468-2667(20)30074-8 32220653 PMC7104094

[4] Miller IF Becker AD Grenfell BT Metcalf CJE (2020). Disease and Healthcare burden of COVID-19 in the United States. Nat Med 26:1212, 7-+. 10.1038/s41591-020-0952-y 32546823

[5] Linares-Fernández S Raguindin PF. Vaccine Development in the SARS-CoV-2 Pandemic: a Balancing Act on Accuracy and Speed. Int J Public Health (2020). 65:1433–4. 10.1007/s00038-020-01511-2 33047152 PMC7550229

[6] Pfefferbaum B North CS. Mental Health and the Covid-19 Pandemic. N Engl J Med (2020). 383:510–2. 10.1056/NEJMp2008017 32283003

[7] Bavel JJV Baicker K Boggio PS Capraro V Cichocka A Cikara M Using Social and Behavioural Science to Support COVID-19 Pandemic Response. Nat Hum Behav (2020). 4:460–71. 10.1038/s41562-020-0884-z 32355299

[8] Kraemer MUG Yang C-H Gutierrez B Wu C-H Klein B Pigott DM (2020). The Effect of Human Mobility and Control Measures on the COVID-19 Epidemic in China. Science 368:493, 7-+. 10.1126/science.abb4218 32213647 PMC7146642

[9] World Health Organisation. Coronavirus Disease (COVID-19) Advice for the Public (2020a). Available at: https://www.who.int/emergencies/diseases/novel-coronavirus-2019/advice-for-public ( Accessed May 10, 2020).

[10] Maier BF Brockmann D (2020). Effective Containment Explains Subexponential Growth in Recent Confirmed COVID-19 Cases in China. Science 368:742, 6-+. 10.1126/science.abb4557 32269067 PMC7164388

[11] World Health Organization. Transmission of SARS-CoV-2: Implications for Infection Prevention Precautions (2020b). Available at: https://www.who.int/news-room/commentaries/detail/transmission-of-sarscov-2-implications-for-infection-prevention-precautions (Accessed July 9, 2020).

[12] Chu DK Akl EA Duda S Solo K Yaacoub S Schünemann HJ Physical Distancing, Face Masks, and Eye protection to Prevent Person-To-Person Transmission of SARS-CoV-2 and COVID-19: a Systematic Review and Meta-Analysis. The Lancet (2020). 395:1973–87. 10.1016/s0140-6736(20)31142-9 PMC7263814 32497510

[13] Yıldırım M Güler A. COVID-19 Severity, Self-Efficacy, Knowledge, Preventive Behaviors, and Mental Health in Turkey. Death Stud (2020). 16:1–8. 10.1080/07481187.2020.1793434 32673183

[14] Childs CE Calder PC Miles EA. Diet and Immune Function. Nutrients (2019). 11:1933. 10.3390/nu11081933 PMC6723551 31426423

[15] Laddu DR Lavie CJ Phillips SA Arena R. Physical Activity for Immunity protection: Inoculating Populations with Healthy Living Medicine in Preparation for the Next Pandemic. Prog Cardiovasc Dis (2021). 64:102–4. 10.1016/j.pcad.2020.04.006 32278694 PMC7195025

[16] Nieman DC Wentz LM. The Compelling Link between Physical Activity and the Body's Defense System. J Sport Health Sci (2019). 8:201–17. 10.1016/j.jshs.2018.09.009 31193280 PMC6523821

[17] World Health Organisation. COVID-19: Vulnerable and High Risk Groups (2020b). Available at: https://www.who.int/westernpacific/emergencies/covid-19/information/high-risk-groups (Accessed May 08, 2020).

[18] Yang J Zheng Y Gou X Pu K Chen Z Guo Q Prevalence of Comorbidities and its Effects in Patients Infected with SARS-CoV-2: a Systematic Review and Meta-Analysis. Int J Infect Dis (2020). 94:91–5. 10.1016/j.ijid.2020.03.017 32173574 PMC7194638

[19] Clark A Jit M Warren-Gash C Guthrie B Wang HHX Mercer SW Global, Regional, and National Estimates of the Population at Increased Risk of Severe COVID-19 Due to Underlying Health Conditions in 2020: a Modelling Study. Lancet Glob Health (2020). 8:e1003–1017. 10.1016/S2214-109x(20)30264-3 32553130 PMC7295519

[20] Harrison SL Fazio-Eynullayeva E Lane DA Underhill P Lip GYH. Comorbidities Associated with Mortality in 31,461 Adults with COVID-19 in the United States: A Federated Electronic Medical Record Analysis. Plos Med (2020). 17:e1003321. 10.1371/journal.pmed.1003321 32911500 PMC7482833

[21] Pasquariello P Stranges S. Excess Mortality from COVID-19: a Commentary on the Italian Experience. Int J Public Health (2020). 65:529–31. 10.1007/s00038-020-01399-y 32468219

[22] Lazzerini M Barbi E Apicella A Marchetti F Cardinale F Trobia G. Delayed Access or Provision of Care in Italy Resulting from Fear of COVID-19. Lancet Child Adolesc Health (2020). 4:E10–E11. 10.1016/S2352-4642(20)30108-5 32278365 PMC7146704

[23] Nguyen TT Le NT Nguyen MH Pham LV Do BN Nguyen HC Health Literacy and Preventive Behaviors Modify the Association between Pre-existing Health Conditions and Suspected COVID-19 Symptoms: A Multi-Institutional Survey. Ijerph (2020c). 17:8598. 10.3390/ijerph17228598 PMC7699410 33228096

[24] Pham KM Pham LV Phan DT Tran TV Nguyen HC Nguyen MH Healthy Dietary Intake Behavior Potentially Modifies the Negative Effect of COVID-19 Lockdown on Depression: A Hospital and Health Center Survey. Front Nutr (2020). 7:230. 10.3389/fnut.2020.581043 PMC7701254 33304917

[25] Prime Minister of Vietnam. Gov’t Extends Social Distancing for at Least One Week in 28 Localities (2020a). Available at: http://news.chinhphu.vn/Home/Govt-extends-social-distancing-for-at-least-one-week-in-28-localities/20204/39735.vgp (Accessed April 20, 2020).

[26] Prime Minister of Vietnam. PM Orders Strict Nationwide Social Distancing Rules. starting April 1 (2020b). Available at: https://vietnamlawmagazine.vn/pm-orders-strict-nationwide-social-distancing-rules-starting-april-1-27108.html (Accessed March 31, 2020).

[27] BMJ Editorial Team. Overview of Novel Coronavirus (2019-nCoV). BMJ Best Practice (2020). Available at: https://bestpractice.bmj.com/topics/en-gb/3000165 (Accessed March 10, 2020).

[28] Charlson ME Pompei P Ales KL MacKenzie CR. A New Method of Classifying Prognostic Comorbidity in Longitudinal Studies: Development and Validation. J Chronic Dis (1987). 40:373–83. 10.1016/0021-9681(87)90171-8 3558716

[29] Duong TV Aringazina A Kayupova G Nurjanah fnm Pham TV Pham KM Development and Validation of a New Short-form Health Literacy Instrument (HLS-SF12) for the General Public in Six Asian Countries. HLRP: Health Literacy Res Pract (2019a). 3:e91–e102. 10.3928/24748307-20190225-01 PMC6607763 31294310

[30] Duong TV Nguyen TTP Pham KM Nguyen KT Giap MH Tran TDX Validation of the Short-form Health Literacy Questionnaire (HLS-SF12) and its Determinants Among People Living in Rural Areas in Vietnam. Ijerph (2019b). 16:3346. 10.3390/ijerph16183346 PMC6765800 31514271

[31] Iacobucci D Posavac SS Kardes FR Schneider MJ Popovich DL. Toward a More Nuanced Understanding of the Statistical Properties of a Median Split. J Consumer Psychol (2015). 25:652–65. 10.1016/j.jcps.2014.12.002

[32] Kroenke K Spitzer RL Williams JBW. The PHQ-9. J Gen Intern Med (2001). 16:606–13. 10.1046/j.1525-1497.2001.016009606.x 11556941 PMC1495268

[33] Ruiz MA Zamorano E García-Campayo J Pardo A Freire O Rejas J. Validity of the GAD-7 Scale as an Outcome Measure of Disability in Patients with Generalized Anxiety Disorders in Primary Care. J Affective Disord (2011). 128:277–86. 10.1016/j.jad.2010.07.010 20692043

[34] Pollack AA Weiss B Trung LT. Mental Health, Life Functioning and Risk Factors Among People Exposed to Frequent Natural Disasters and Chronic Poverty in Vietnam. BJPsych open (2016). 2:221–32. 10.1192/bjpo.bp.115.002170 27458524 PMC4957519

[35] Kroenke K Spitzer RL Williams JBW Monahan PO Löwe B. Anxiety Disorders in Primary Care: Prevalence, Impairment, Comorbidity, and Detection. Ann Intern Med (2007). 146:317–25. 10.7326/0003-4819-146-5-200703060-00004 17339617

[36] National Center for Immunization and Respiratory Diseases. What Healthcare Personnel Should Know about Caring for Patients with Confirmed or Possible 2019-nCoV Infection (2020). Available at: https://www.cdc.gov/coronavirus/2019-ncov/hcp/caringfor-patients.html (Accessed June 15, 2020).

[37] World Health Organization. Novel Coronavirus (2019-nCoV) Technical Guidance (2020a). Available at: https://www.who.int/emergencies/diseases/novel-coronavirus-2019/technicalguidance (Accessed April 15, 2020).

[38] Mazza C Ricci E Biondi S Colasanti M Ferracuti S Napoli C A Nationwide Survey of Psychological Distress Among Italian People during the COVID-19 Pandemic: Immediate Psychological Responses and Associated Factors. Ijerph (2020). 17:3165. 10.3390/ijerph17093165 PMC7246819 32370116

[39] Wang C Pan R Wan X Tan Y Xu L Ho CS Immediate Psychological Responses and Associated Factors during the Initial Stage of the 2019 Coronavirus Disease (COVID-19) Epidemic Among the General Population in China. Ijerph (2020a). 17:1729. 10.3390/ijerph17051729 PMC7084952 32155789

[40] Østergaard SD Foldager L. The Association between Physical Illness and Major Depressive Episode in General Practice. Acta Psychiatr Scand (2011). 123:290–6. 10.1111/j.1600-0447.2010.01668.x 21219268

[41] Jain V Yuan J-M. Predictive Symptoms and Comorbidities for Severe COVID-19 and Intensive Care Unit Admission: a Systematic Review and Meta-Analysis. Int J Public Health (2020). 65:533–46. 10.1007/s00038-020-01390-7 32451563 PMC7246302

[42] Giallonardo V Sampogna G Del Vecchio V Luciano M Albert U Carmassi C The Impact of Quarantine and Physical Distancing Following COVID-19 on Mental Health: Study Protocol of a Multicentric Italian Population Trial. Front Psychiatry (2020). 11. 10.3389/fpsyt.2020.00533 PMC7290062 32581895

[43] Chew NWS Lee GKH Tan BYQ Jing M Goh Y Ngiam NJH A Multinational, Multicentre Study on the Psychological Outcomes and Associated Physical Symptoms Amongst Healthcare Workers during COVID-19 Outbreak. Brain Behav Immun (2020). 88:559–65. 10.1016/j.bbi.2020.04.049 32330593 PMC7172854

[44] Nguyen HC Nguyen MH Do BN Tran CQ Nguyen TTP Pham KM People with Suspected COVID-19 Symptoms Were More Likely Depressed and Had Lower Health-Related Quality of Life: The Potential Benefit of Health Literacy. Jcm (2020a). 9:965. 10.3390/jcm9040965 PMC7231234 32244415

[45] Wang C Pan R Wan X Tan Y Xu L McIntyre RS A Longitudinal Study on the Mental Health of General Population during the COVID-19 Epidemic in China. Brain Behav Immun (2020b). 87:40–8. 10.1016/j.bbi.2020.04.028 32298802 PMC7153528

[46] Galea S Merchant RM Lurie N. The Mental Health Consequences of COVID-19 and Physical Distancing. JAMA Intern Med (2020). 180:817–8. 10.1001/jamainternmed.2020.1562 32275292

[47] Loftus MJ Guitart C Tartari E Stewardson AJ Amer F Bellissimo-Rodrigues F Hand hygiene in Low- and Middle-Income Countries. Int J Infect Dis (2019). 86:25–30. 10.1016/j.ijid.2019.06.002 31189085

[48] Fletcher GF Landolfo C Niebauer J Ozemek C Arena R Lavie CJ. Promoting Physical Activity and Exercise. J Am Coll Cardiol (2018). 72:1622–39. 10.1016/j.jacc.2018.08.2141 30522636

[49] Lavie CJ Lee D-c. Sui X Arena R O'Keefe JH Church TS Effects of Running on Chronic Diseases and Cardiovascular and All-Cause Mortality. Mayo Clinic Proc (2015). 90:1541–52. 10.1016/j.mayocp.2015.08.001 26362561

[50] Kvam S Kleppe CL Nordhus IH Hovland A. Exercise as a Treatment for Depression: A Meta-Analysis. J Affective Disord (2016). 202:67–86. 10.1016/j.jad.2016.03.063 27253219

[51] Wei K Nyunt MSZ Gao Q Wee SL Ng TP. Long-term Changes in Nutritional Status Are Associated with Functional and Mortality Outcomes Among Community-Living Older Adults. Nutrition (2019). 66:180–6. 10.1016/j.nut.2019.05.006 31310959

[52] Li Y Lv M-R Wei Y-J Sun L Zhang J-X Zhang H-G Dietary Patterns and Depression Risk: A Meta-Analysis. Psychiatry Res (2017). 253:373–82. 10.1016/j.psychres.2017.04.020 28431261

[53] Molendijk M Molero P Ortuño Sánchez-Pedreño F Van der Does W Angel Martínez-González M. Diet Quality and Depression Risk: A Systematic Review and Dose-Response Meta-Analysis of Prospective Studies. J Affective Disord (2018). 226:346–54. 10.1016/j.jad.2017.09.022 29031185

[54] Gasmi A Noor S Tippairote T Dadar M Menzel A Bjørklund G. Individual Risk Management Strategy and Potential Therapeutic Options for the COVID-19 Pandemic. Clin Immunol (2020). 215:108409. 10.1016/j.clim.2020.108409 32276137 PMC7139252

[55] Naja F Hamadeh R. Nutrition amid the COVID-19 Pandemic: a Multi-Level Framework for Action. Eur J Clin Nutr (2020). 74:1117–21. 10.1038/s41430-020-0634-3 32313188 PMC7167535

[56] Ranasinghe C Ozemek C Arena R. Exercise and Well-Being during COVID 19 - Time to Boost Your Immunity. Expert Rev Anti-infective Ther (2020). 18:1195–200. 10.1080/14787210.2020.1794818 32662717

[57] Do BN Nguyen P-A Pham KM Nguyen HC Nguyen MH Tran CQ Determinants of Health Literacy and its Associations with Health-Related Behaviors, Depression Among the Older People with and without Suspected COVID-19 Symptoms: A Multi-Institutional Study. Front Public Health (2020). 8:694. 10.3389/fpubh.2020.581746 PMC7703185 33313037

[58] Duong TV Pham KM Do BN Kim GB Dam HTB Le V-TT Digital Healthy Diet Literacy and Self-Perceived Eating Behavior Change during COVID-19 Pandemic Among Undergraduate Nursing and Medical Students: A Rapid Online Survey. Ijerph (2020). 17:7185. 10.3390/ijerph17197185 PMC7579441 33008102

[59] Mathiesen EF Nome S Eisemann M Richter J. Drinking Patterns, Psychological Distress and Quality of Life in a Norwegian General Population-Based Sample. Qual Life Res (2012). 21:1527–36. 10.1007/s11136-011-0080-8 22219172

[60] Nguyen HT Do BN Pham KM Kim GB Dam HTB Nguyen TT Fear of COVID-19 Scale-Associations of its Scores with Health Literacy and Health-Related Behaviors Among Medical Students. Ijerph (2020b). 17:4164. 10.3390/ijerph17114164 PMC7311979 32545240

[61] Rodriguez LM Litt DM Stewart SH. Drinking to Cope with the Pandemic: The Unique Associations of COVID-19-Related Perceived Threat and Psychological Distress to Drinking Behaviors in American Men and Women. Addict Behaviors (2020). 110:106532. 10.1016/j.addbeh.2020.106532 PMC7320671 32652385

[62] Wardell JD Kempe T Rapinda KK Single A Bilevicius E Frohlich JR Drinking to Cope during COVID‐19 Pandemic: The Role of External and Internal Factors in Coping Motive Pathways to Alcohol Use, Solitary Drinking, and Alcohol Problems. Alcohol Clin Exp Res (2020). 44:2073–83. 10.1111/acer.14425 32870516

[63] Ivan MC Amspoker AB Nadorff MR Kunik ME Cully JA Wilson N Alcohol Use, Anxiety, and Insomnia in Older Adults with Generalized Anxiety Disorder. Am J Geriatr Psychiatry (2014). 22:875–83. 10.1016/j.jagp.2013.04.001 23973253 PMC3842378

[64] MacAndrew C. An Examination of the Relevance of the Individual Differences (A-Trait) Formulation of the Tension-Reduction Theory to the Etiology of Alcohol Abuse in Young Males. Addict Behaviors (1982). 7:39–45. 10.1016/0306-4603(82)90023-5 7080883

[65] Churchill SA Farrell L. Alcohol and Depression: Evidence from the 2014 Health Survey for England. Drug Alcohol Depend (2017). 180:86–92. 10.1016/j.drugalcdep.2017.08.006 28886396

[66] Rehm J Gmel GE Gmel G Hasan OSM Imtiaz S Popova S The Relationship between Different Dimensions of Alcohol Use and the burden of Disease-An Update. Addiction (2017). 112:968–1001. 10.1111/add.13757 28220587 PMC5434904

[67] Vietnam Ministry of Health. Coronavirus Disease (COVID-19) Outbreak in Vietnam (2020). Available at: https://ncov.moh.gov.vn/ (Accessed June 07, 2020).

