# Supplementary material for: Associations of Underlying Health Conditions With Anxiety and Depression Among Outpatients: Modification Effects of Suspected COVID-19 Symptoms, Health-Related and Preventive Behaviors
Source: Int J Public Health. 2021 Jun 23;66:634904. doi: 10.3389/ijph.2021.634904 (PMC8284590; doi:10.3389/ijph.2021.634904)
Supplement: Supplementary file 1 [file Datasheet1.docx]

**International Journal of Public Health**

**Supplementary Material**

**Table S1** Spearman’s correlations among confounders of anxiety, Vietnam, 2020 (n= 4348)

|  | Age | Gender | Marital status | Education | Occupation | Ability to pay | Social status | Lockdown | BMI |
| --- | --- | --- | --- | --- | --- | --- | --- | --- | --- |
| Gender | 0.09 |  |  |  |  |  |  |  |  |
| Marital status | **0.35** | -0.03 |  |  |  |  |  |  |  |
| Education | **-0.43** | 0.01 | -0.25 |  |  |  |  |  |  |
| Occupation | -0.14 | 0.02 | 0.06 | 0.16 |  |  |  |  |  |
| Ability to pay | -0.22 | -0.03 | -0.06 | 0.26 | 0.17 |  |  |  |  |
| Social status | -0.08 | 0.00 | 0.01 | 0.24 | 0.18 | **0.30** |  |  |  |
| Lockdown | 0.15 | 0.08 | 0.15 | -0.09 | -0.02 | -0.16 | -0.04 |  |  |
| BMI | 0.00 | 0.06 | 0.06 | -0.01 | 0.01 | 0.04 | 0.03 | -0.03 |  |
| HL | **-0.32** | -0.02 | -0.18 | 0.19 | 0.08 | 0.19 | 0.18 | -0.04 | 0.02 |

**Abbreviations** BMI, body mass index; HL, health literacy

**Table S2** Spearman’s correlations among confounders of depression, Vietnam, 2020 (n= 8291)

|  | Age | Gender | Marital status | Education | Occupation | Ability to pay | Social status | Lockdown | BMI |
| --- | --- | --- | --- | --- | --- | --- | --- | --- | --- |
| Gender | 0.03 |  |  |  |  |  |  |  |  |
| Marital status | **0.37** | -0.06 |  |  |  |  |  |  |  |
| Education | **-0.40** | 0.03 | -0.26 |  |  |  |  |  |  |
| Occupation | -0.13 | 0.01 | 0.02 | 0.12 |  |  |  |  |  |
| Ability to pay | -0.17 | -0.01 | -0.08 | 0.18 | 0.16 |  |  |  |  |
| Social status | -0.10 | 0.02 | -0.00 | 0.21 | 0.17 | 0.28 |  |  |  |
| Lockdown | 0.06 | 0.01 | 0.11 | 0.00 | -0.09 | -0.19 | -0.10 |  |  |
| BMI | 0.02 | 0.06 | 0.06 | -0.00 | 0.00 | 0.03 | 0.03 | -0.00 |  |
| HL | **-0.31** | 0.03 | -0.19 | 0.22 | 0.12 | 0.25 | 0.22 | -0.12 | 0.01 |

**Abbreviations** BMI, body mass index; HL, health literacy

**Table S3** Factors associated with depression and anxiety among study participants using bivariate logistic models, Vietnam, 2020

| **Variables** | **Depression (PHQ ≥ 10)** | | **Anxiety (GAD ≥ 8) *** | |
| --- | --- | --- | --- | --- |
|  | **OR (95% CI)** | ***p*** | **OR (95% CI)** | ***p*** |
| **Confounders** |  |  |  |  |
| Age groups |  |  |  |  |
| 18 – 39 | 1.00 |  | 1.00 |  |
| 40 – 59 | 1.57 (1.32, 1.88) | < 0.001 | 2.04 (1.62, 2.57) | <0.001 |
| ≥ 60 | 5.25 (4.47, 6.16) | < 0.001 | 9.93 (8.06, 12.24) | <0.001 |
| Gender |  |  |  |  |
| Women | 1.00 |  |  |  |
| Men | 0.99 (0.87, 1.13) | 0.906 | 1.12 (0.95, 1.31) | 0.182 |
| Marital status |  |  |  |  |
| Never married | 1.00 |  |  |  |
| Ever married | 1.68 (1.39, 2.02) | < 0.001 | 2.57 (1.96, 3.37) | <0.001 |
| Education attainment |  |  |  |  |
| Secondary school or below | 1.00 |  | 1.00 |  |
| High school | 0.87 (0.73, 1.03) | 0.102 | 0.75 (0.61, 0.93) | 0.009 |
| College/university or higher | 0.81 (0.69, 0.94) | 0.007 | 0.57 (0.47, 0.69) | <0.001 |
| Occupation |  |  |  |  |
| Unemployed/ Dependents | 1.00 |  | 1.00 |  |
| Employed | 0.39 (0.32, 0.47) | <0.001 | 0.51 (0.41, 0.64) | <0.001 |
| Ability to pay for treatments |  |  |  |  |
| Very or fairly difficult | 1.00 |  | 1.00 |  |
| Very or fairly easy | 0.37 (0.32, 0.43) | < 0.001 | 0.30 (0.25, 0.38) | <0.001 |
| Social status |  |  |  |  |
| Low | 1.00 |  | 1.00 |  |
| Middle or high | 0.74 (0.63, 0.87) | < 0.001 | 1.47 (1.19, 1.82) | <0.001 |
| Lockdown measure |  |  |  |  |
| No | 1.00 |  | 1.00 |  |
| Yes | 3.33 (2.91, 3.80) | < 0.001 | 2.89 (2.42, 3.46) | <0.001 |
| BMI, kg/m^2^ |  |  |  |  |
| Normal weight (BMI < 25.0) | 1.00 |  | 1.00 |  |
| Overweight/obese (BMI ≥ 25.0) | 1.00 (0.82, 1.22) | 0.999 | 1.06 (0.84, 1.35) | 0.616 |
| HL score, 1 score increment | 0.95 (0.94, 0.96) | < 0.001 | 0.95 (0.94, 0.96) | <0.001 |

**Abbreviations** PHQ, patient health questionnaire; GAD, Generalized Anxiety Disorder; OR, odd ratio; CI, confidence interval; S-COVID-19-S, suspected corona virus disease-2019 symptoms; BMI, body mass index; PB, protective behaviors; HL, health literacy.

* Anxiety was analyzed on the sample of 4348 participants

^a^ The suspected COVID-19 symptoms including common symptom (fever, cough, dyspnea), less common symptom (myalgia, fatigue, sputum production, confusion, headache, sore throat, rhinorrhea, chest pain, hemoptysis, diarrhea, and nausea/vomiting).

^b^ People were asked whether their health-related behaviors are getting worse, better, or unchanged during COVID-19 pandemic as compared to those before the pandemic.

**Table S4** Associations of underlying health conditions with depression, and anxiety using logistic regression models, Vietnam, 2020

| **Variable** | **Depression (PHQ ≥ 10)** | | | | **Anxiety (GAD ≥ 8) *** | | | |
| --- | --- | --- | --- | --- | --- | --- | --- | --- |
|  | **Model 1** | | **Model 2** | | **Model 1** | | **Model 2** | |
|  | **OR (95% CI)** | ***p*** | **OR (95% CI)** | ***p*** | **OR (95% CI)** | ***p*** | **OR (95% CI)** | ***p*** |
| Underlying health conditions |  |  |  |  |  |  |  |  |
| Non-UHC | 1.00 |  | 1.00 |  | 1.00 |  | 1.00 |  |
| UHC | 3.94 (3.44, 4.51) | <0.001 | 2.11 (1.81, 2.46) | <0.001 | 4.94 (4.18, 5.83) | <0.001 | 2.86 (2.37, 3.46) | <0.001 |
| Age groups |  |  |  |  |  |  |  |  |
| 18 – 39 | 1.00 |  | 1.00 |  | 1.00 |  | 1.00 |  |
| 40 – 59 | 1.57 (1.32, 1.88) | <0.001 | 1.36 (1.12, 1.63) | 0.001 | 2.04 (1.62, 2.57) | <0.001 | 1.69 (1.33, 2.15) | <0.001 |
| ≥ 60 | 5.25 (4.47, 6.16) | <0.001 | 3.45 (2.89, 4.12) | <0.001 | 9.93 (8.06, 12.2) | <0.001 | 6.44 (5.14, 8.08) | <0.001 |
| Gender |  |  |  |  |  |  |  |  |
| Women | 1.00 |  | 1.00 |  | 1.00 |  | 1.00 |  |
| Men | 0.99 (0.87, 1.13) | 0.906 | 0.91 (0.78, 1.05) | 0.178 | 1.12 (0.95, 1.31) | 0.182 | 0.86 (0.72, 1.04) | 0.122 |
| Occupation |  |  |  |  |  |  |  |  |
| Unemployed/ Dependents | 1.00 |  | 1.00 |  | 1.00 |  | 1.00 |  |
| Employed | 0.39 (0.32, 0.47) | <0.001 | 0.69 (0.56, 0.86) | 0.001 | 0.51 (0.41, 0.64) | <0.001 | 0.71 (0.54, 0.92) | 0.011 |
| Ability to pay for medication |  |  |  |  |  |  |  |  |
| Very or fairly difficult | 1.00 |  | 1.00 |  |  |  |  |  |
| Very or fairly easy | 0.37 (0.32, 0.43) | <0.001 | 0.57 (0.48, 0.67) | <0.001 |  |  |  |  |
| Social status |  |  |  |  |  |  |  |  |
| Low | 1.00 |  | 1.00 |  | 1.00 |  | 1.00 |  |
| Middle or high | 0.74 (0.63, 0.87) | <0.001 | 1.22 (1.01, 1.46) | 0.034 | 1.47 (1.19, 1.82) | <0.001 | 2.03 (1.60, 2.58) | <0.001 |
| Lockdown measure |  |  |  |  |  |  |  |  |
| No | 1.00 |  | 1.00 |  | 1.00 |  | 1.00 |  |
| Yes | 3.33 (2.91, 3.80) | <0.001 | 2.45 (2.12, 2.83) | <0.001 | 2.89 (2.42, 3.46) | <0.001 | 1.95 (1.60, 2.37) | <0.001 |

**Abbreviations** PHQ, patient health questionnaire; GAD, Generalized Anxiety Disorder; OR, odd ratio; CI, confidence interval; UHC, underlying health conditions.

**^*^** Anxiety was analyzed on the sample of 4348 participants

**Table S5** Associations of S-COVID-19-S with depression, and anxiety using logistic regression models, Vietnam, 2020

| **Variable** | **Depression (PHQ ≥ 10)** | | | | **Anxiety (GAD ≥ 8) *** | | | |
| --- | --- | --- | --- | --- | --- | --- | --- | --- |
|  | **Model 1** | | **Model 2** | | **Model 1** | | **Model 2** | |
|  | **OR (95% CI)** | ***p*** | **OR (95% CI)** | ***p*** | **OR (95% CI)** | ***p*** | **OR (95% CI)** | ***p*** |
| S-COVID-19-S |  |  |  |  |  |  |  |  |
| No | 1.00 |  | 1.00 |  | 1.00 |  | 1.00 |  |
| Yes | 4.14 (3.60, 4.75) | <0.001 | 2.84 (2.45, 3.29) | <0.001 | 4.41 (3.71, 5.24) | <0.001 | 2.92 (2.42, 3.52) | <0.001 |
| Age groups |  |  |  |  |  |  |  |  |
| 18 – 39 | 1.00 |  | 1.00 |  | 1.00 |  | 1.00 |  |
| 40 – 59 | 1.57 (1.32, 1.88) | <0.001 | 1.36 (1.13, 1.64) | 0.001 | 2.04 (1.62, 2.57) | <0.001 | 1.86 (1.47, 2.36) | <0.001 |
| ≥ 60 | 5.25 (4.47, 6.16) | <0.001 | 3.50 (2.94, 4.17) | <0.001 | 9.93 (8.06, 12.2) | <0.001 | 7.18 (5.74, 8.98) | <0.001 |
| Gender |  |  |  |  |  |  |  |  |
| Women | 1.00 |  | 1.00 |  | 1.00 |  | 1.00 |  |
| Men | 0.99 (0.87, 1.13) | 0.906 | 0.92 (0.80, 1.07) | 0.285 | 1.12 (0.95, 1.31) | 0.182 | 0.92 (0.76, 1.10) | 0.356 |
| Occupation |  |  |  |  |  |  |  |  |
| Unemployed/ Dependents | 1.00 |  | 1.00 |  | 1.00 |  | 1.00 |  |
| Employed | 0.39 (0.32, 0.47) | <0.001 | 0.71 (0.56, 0.88) | 0.002 | 0.51 (0.41, 0.64) | <0.001 | 0.74 (0.57, 0.96) | 0.026 |
| Ability to pay for medication |  |  |  |  |  |  |  |  |
| Very or fairly difficult | 1.00 |  | 1.00 |  |  |  |  |  |
| Very or fairly easy | 0.37 (0.32, 0.43) | <0.001 | 0.63 (0.54, 0.75) | <0.001 |  |  |  |  |
| Social status |  |  |  |  |  |  |  |  |
| Low | 1.00 |  | 1.00 |  | 1.00 |  | 1.00 |  |
| Middle or high | 0.74 (0.63, 0.87) | <0.001 | 1.15 (0.96, 1.38) | 0.133 | 1.47 (1.19, 1.82) | <0.001 | 1.88 (1.48, 2.39) | <0.001 |
| Lockdown measure |  |  |  |  |  |  |  |  |
| No | 1.00 |  | 1.00 |  | 1.00 |  | 1.00 |  |
| Yes | 3.33 (2.91, 3.80) | <0.001 | 2.61 (2.26, 3.02) | <0.001 | 2.89 (2.42, 3.46) | <0.001 | 1.97 (1.62, 2.40) | <0.001 |

**Abbreviations** PHQ, patient health questionnaire; GAD, Generalized Anxiety Disorder; S-COVID-19-S, suspected corona virus disease-2019 symptoms; OR, odd ratio; CI, confidence interval.

**^*^** Anxiety was analyzed on the sample of 4348 participants.

**Table S6** Associations of smoking with depression, and anxiety using logistic regression models, Vietnam, 2020

| **Variable** | **Depression (PHQ ≥ 10)** | | | | **Anxiety (GAD ≥ 8) *** | | | |
| --- | --- | --- | --- | --- | --- | --- | --- | --- |
|  | **Model 1** | | **Model 2** | | **Model 1** | | **Model 2** | |
|  | **OR (95% CI)** | ***p*** | **OR (95% CI)** | ***p*** | **OR (95% CI)** | ***p*** | **OR (95% CI)** | ***p*** |
| Smoking |  |  |  |  |  |  |  |  |
| Never, stopped, or smoke less | 1.00 |  | 1.00 |  | 1.00 |  | 1.00 |  |
| Unchanged or smoke more | 1.04 (0.86, 1.25) | 0.680 | 1.08 (0.84, 1.38) | 0.550 | 1.57 (1.19, 2.07) | 0.001 | 1.02 (0.73, 1.42) | 0.895 |
| Age groups |  |  |  |  |  |  |  |  |
| 18 – 39 | 1.00 |  | 1.00 |  | 1.00 |  | 1.00 |  |
| 40 – 59 | 1.57 (1.32, 1.88) | <0.001 | 1.53 (1.27, 1.83) | <0.001 | 2.04 (1.62, 2.57) | <0.001 | 2.05 (1.62, 2.59) | <0.001 |
| ≥ 60 | 5.25 (4.47, 6.16) | <0.001 | 4.42 (3.74, 5.23) | <0.001 | 9.93 (8.06, 12.2) | <0.001 | 9.06 (7.29, 11.2) | <0.001 |
| Gender |  |  |  |  |  |  |  |  |
| Women | 1.00 |  | 1.00 |  | 1.00 |  | 1.00 |  |
| Men | 0.99 (0.87, 1.13) | 0.906 | 0.91 (0.79, 1.05) | 0.206 | 1.12 (0.95, 1.31) | 0.182 | 0.89 (0.74, 1.07) | 0.226 |
| Occupation |  |  |  |  |  |  |  |  |
| Unemployed/ Dependents | 1.00 |  | 1.00 |  | 1.00 |  | 1.00 |  |
| Employed | 0.39 (0.32, 0.47) | <0.001 | 0.69 (0.55, 0.85) | 0.001 | 0.51 (0.41, 0.64) | <0.001 | 0.70 (0.54, 0.91) | 0.008 |
| Ability to pay for medication |  |  |  |  |  |  |  |  |
| Very or fairly difficult | 1.00 |  | 1.00 |  |  |  |  |  |
| Very or fairly easy | 0.37 (0.32, 0.43) | <0.001 | 0.56 (0.48, 0.66) | <0.001 |  |  |  |  |
| Social status |  |  |  |  |  |  |  |  |
| Low | 1.00 |  | 1.00 |  | 1.00 |  | 1.00 |  |
| Middle or high | 0.74 (0.63, 0.87) | <0.001 | 1.20 (1.01, 1.44) | 0.046 | 1.47 (1.19, 1.82) | <0.001 | 1.95 (1.54, 2.47) | <0.001 |
| Lockdown measure |  |  |  |  |  |  |  |  |
| No | 1.00 |  | 1.00 |  | 1.00 |  | 1.00 |  |
| Yes | 3.33 (2.91, 3.80) | <0.001 | 2.88 (2.50, 3.32) | <0.001 | 2.89 (2.42, 3.46) | <0.001 | 2.37 (1.95, 2.87) | <0.001 |

**Abbreviations:** PHQ, patient health questionnaire; GAD, Generalized Anxiety Disorder; OR, odd ratio; CI, confidence interval.

**^*^** Anxiety was analyzed on the sample of 4348 participants

**Table S7** Associations of drinking alcohol with depression, and anxiety using logistic regression models, Vietnam, 2020

| **Variable** | **Depression (PHQ ≥ 10)** | | | | **Anxiety (GAD ≥ 8) *** | | | |
| --- | --- | --- | --- | --- | --- | --- | --- | --- |
|  | **Model 1** | | **Model 2** | | **Model 1** | | **Model 2** | |
|  | **OR (95% CI)** | ***p*** | **OR (95% CI)** | ***p*** | **OR (95% CI)** | ***p*** | **OR (95% CI)** | ***p*** |
| Drinking alcohol |  |  |  |  |  |  |  |  |
| Never, stopped, or drink less | 1.00 |  | 1.00 |  | 1.00 |  | 1.00 |  |
| Unchanged or drink more | 1.04 (0.86, 1.25) | 0.680 | 1.19 (0.98, 1.45) | 0.081 | 1.57 (1.19, 2.07) | 0.001 | 1.99 (1.45, 2.72) | <0.001 |
| Age groups |  |  |  |  |  |  |  |  |
| 18 – 39 | 1.00 |  | 1.00 |  | 1.00 |  | 1.00 |  |
| 40 – 59 | 1.57 (1.32, 1.88) | <0.001 | 1.52 (1.26, 1.82) | <0.001 | 2.04 (1.62, 2.57) | <0.001 | 2.02 (1.59, 2.55) | <0.001 |
| ≥ 60 | 5.25 (4.47, 6.16) | <0.001 | 4.41 (3.72, 5.22) | <0.001 | 9.93 (8.06, 12.2) | <0.001 | 8.86 (7.12, 11.1) | <0.001 |
| Gender |  |  |  |  |  |  |  |  |
| Women | 1.00 |  | 1.00 |  | 1.00 |  | 1.00 |  |
| Men | 0.99 (0.87, 1.13) | 0.906 | 0.83 (0.71, 0.96) | 0.012 | 1.12 (0.95, 1.31) | 0.182 | 0.82 (0.68, 0.99) | 0.035 |
| Occupation |  |  |  |  |  |  |  |  |
| Unemployed/ Dependents | 1.00 |  | 1.00 |  | 1.00 |  | 1.00 |  |
| Employed | 0.39 (0.32, 0.47) | <0.001 | 0.67 (0.54, 0.84) | <0.001 | 0.51 (0.41, 0.64) | <0.001 | 0.71 (0.55, 0.92) | 0.010 |
| Ability to pay for medication |  |  |  |  |  |  |  |  |
| Very or fairly difficult | 1.00 |  | 1.00 |  |  |  |  |  |
| Very or fairly easy | 0.37 (0.32, 0.43) | <0.001 | 0.56 (0.48, 0.66) | <0.001 |  |  |  |  |
| Social status |  |  |  |  |  |  |  |  |
| Low | 1.00 |  | 1.00 |  | 1.00 |  | 1.00 |  |
| Middle or high | 0.74 (0.63, 0.87) | <0.001 | 1.20 (1.00, 1.43) | 0.050 | 1.47 (1.19, 1.82) | <0.001 | 1.97 (1.56, 2.50) | <0.001 |
| Lockdown measure |  |  |  |  |  |  |  |  |
| No | 1.00 |  | 1.00 |  | 1.00 |  | 1.00 |  |
| Yes | 3.33 (2.91, 3.80) | <0.001 | 3.04 (2.63, 3.52) | <0.001 | 2.89 (2.42, 3.46) | <0.001 | 2.47 (2.03, 2.99) | <0.001 |

**Abbreviations** PHQ, patient health questionnaire; GAD, Generalized Anxiety Disorder; OR, odd ratio; CI, confidence interval.

**^*^** Anxiety was analyzed on the sample of 4348 participants

**Table S8** Associations of physical activity with depression, and anxiety using logistic regression models, Vietnam, 2020

| **Variable** | **Depression (PHQ ≥ 10)** | | | | **Anxiety (GAD ≥ 8) *** | | | |
| --- | --- | --- | --- | --- | --- | --- | --- | --- |
|  | **Model 1** | | **Model 2** | | **Model 1** | | **Model 2** | |
|  | **OR (95% CI)** | ***p*** | **OR (95% CI)** | ***p*** | **OR (95% CI)** | ***p*** | **OR (95% CI)** | ***p*** |
| Physical activity |  |  |  |  |  |  |  |  |
| Never, stopped, or exercise less | 1.00 |  | 1.00 |  | 1.00 |  | 1.00 |  |
| Unchanged or exercise more | 0.33 (0.28, 0.37) | <0.001 | 0.53 (0.45, 0.61) | <0.001 | 0.36 (0.30, 0.44) | <0.001 | 0.48 (0.39, 0.59) | <0.001 |
| Age groups |  |  |  |  |  |  |  |  |
| 18 – 39 | 1.00 |  | 1.00 |  | 1.00 |  | 1.00 |  |
| 40 – 59 | 1.57 (1.32, 1.88) | <0.001 | 1.55 (1.29, 1.86) | <0.001 | 2.04 (1.62, 2.57) | <0.001 | 2.03 (1.60, 2.56) | <0.001 |
| ≥ 60 | 5.25 (4.47, 6.16) | <0.001 | 4.30 (3.63, 5.09) | <0.001 | 9.93 (8.06, 12.2) | <0.001 | 8.23 (6.61, 10.3) | <0.001 |
| Gender |  |  |  |  |  |  |  |  |
| Women | 1.00 |  | 1.00 |  | 1.00 |  | 1.00 |  |
| Men | 0.99 (0.87, 1.13) | 0.906 | 0.95 (0.82, 1.09) | 0.465 | 1.12 (0.95, 1.31) | 0.182 | 0.92 (0.76, 1.09) | 0.344 |
| Occupation |  |  |  |  |  |  |  |  |
| Unemployed/ Dependents | 1.00 |  | 1.00 |  | 1.00 |  | 1.00 |  |
| Employed | 0.39 (0.32, 0.47) | <0.001 | 0.71 (0.57, 0.88) | 0.002 | 0.51 (0.41, 0.64) | <0.001 | 0.71 (0.54, 0.92) | 0.009 |
| Ability to pay for medication |  |  |  |  |  |  |  |  |
| Very or fairly difficult | 1.00 |  | 1.00 |  |  |  |  |  |
| Very or fairly easy | 0.37 (0.32, 0.43) | <0.001 | 0.60 (0.51, 0.71) | <0.001 |  |  |  |  |
| Social status |  |  |  |  |  |  |  |  |
| Low | 1.00 |  | 1.00 |  | 1.00 |  | 1.00 |  |
| Middle or high | 0.74 (0.63, 0.87) | <0.001 | 1.21 (1.01, 1.45) | 0.040 | 1.47 (1.19, 1.82) | <0.001 | 1.96 (1.54, 2.48) | <0.001 |
| Lockdown measure |  |  |  |  |  |  |  |  |
| No | 1.00 |  | 1.00 |  | 1.00 |  | 1.00 |  |
| Yes | 3.33 (2.91, 3.80) | <0.001 | 2.23 (1.91, 2.59) | <0.001 | 2.89 (2.42, 3.46) | <0.001 | 2.22 (1.83, 2.69) | <0.001 |

**Abbreviations:** PHQ, patient health questionnaire; GAD, Generalized Anxiety Disorder; OR, odd ratio; CI, confidence interval.

**^*^** Anxiety was analyzed on the sample of 4348 participants

**Table S9** Associations of eating behavior with depression, and anxiety using logistic regression models, Vietnam, 2020

| **Variable** | **Depression (PHQ ≥ 10)** | | | | **Anxiety (GAD ≥ 8) *** | | | |
| --- | --- | --- | --- | --- | --- | --- | --- | --- |
|  | **Model 1** | | **Model 2** | | **Model 1** | | **Model 2** | |
|  | **OR (95% CI)** | ***p*** | **OR (95% CI)** | ***p*** | **OR (95% CI)** | ***p*** | **OR (95% CI)** | ***p*** |
| Eating behavior |  |  |  |  |  |  |  |  |
| Less healthy | 1.00 |  | 1.00 |  | 1.00 |  | 1.00 |  |
| Unchanged or healthier | 0.12 (0.09, 0.15) | <0.001 | 0.22 (0.17, 0.28) | <0.001 | 0.12 (0.10, 0.16) | <0.001 | 0.20 (0.15, 0.26) | <0.001 |
| Age groups |  |  |  |  |  |  |  |  |
| 18 – 39 | 1.00 |  | 1.00 |  | 1.00 |  | 1.00 |  |
| 40 – 59 | 1.57 (1.32, 1.88) | <0.001 | 1.52 (1.26, 1.83) | <0.001 | 2.04 (1.62, 2.57) | <0.001 | 2.01 (1.58, 2.55) | <0.001 |
| ≥ 60 | 5.25 (4.47, 6.16) | <0.001 | 4.06 (3.42, 4.82) | <0.001 | 9.93 (8.06, 12.2) | <0.001 | 7.99 (6.39, 9.99) | <0.001 |
| Gender |  |  |  |  |  |  |  |  |
| Women | 1.00 |  | 1.00 |  | 1.00 |  | 1.00 |  |
| Men | 0.99 (0.87, 1.13) | 0.906 | 0.91 (0.79, 1.06) | 0.226 | 1.12 (0.95, 1.31) | 0.182 | 0.88 (0.73, 1.06) | 0.177 |
| Occupation |  |  |  |  |  |  |  |  |
| Unemployed/ Dependents | 1.00 |  | 1.00 |  | 1.00 |  | 1.00 |  |
| Employed | 0.39 (0.32, 0.47) | <0.001 | 0.71 (0.57, 0.89) | 0.003 | 0.51 (0.41, 0.64) | <0.001 | 0.71 (0.54, 0.93) | 0.012 |
| Ability to pay for medication |  |  |  |  |  |  |  |  |
| Very or fairly difficult | 1.00 |  | 1.00 |  |  |  |  |  |
| Very or fairly easy | 0.37 (0.32, 0.43) | <0.001 | 0.58 (0.49, 0.69) | <0.001 |  |  |  |  |
| Social status |  |  |  |  |  |  |  |  |
| Low | 1.00 |  | 1.00 |  | 1.00 |  | 1.00 |  |
| Middle or high | 0.74 (0.63, 0.87) | <0.001 | 1.13 (0.94, 1.36) | 0.182 | 1.47 (1.19, 1.82) | <0.001 | 1.86 (1.47, 2.37) | <0.001 |
| Lockdown measure |  |  |  |  |  |  |  |  |
| No | 1.00 |  | 1.00 |  | 1.00 |  | 1.00 |  |
| Yes | 3.33 (2.91, 3.80) | <0.001 | 2.44 (2.11, 2.83) | <0.001 | 2.89 (2.42, 3.46) | <0.001 | 2.06 (1.70, 2.51) | <0.001 |

**Abbreviations** PHQ, patient health questionnaire; GAD, Generalized Anxiety Disorder; OR, odd ratio; CI, confidence interval.

**^*^** Anxiety was analyzed on the sample of 4348 participants

**Table S10** Associations of preventive behaviors with depression, and anxiety using logistic regression models, Vietnam, 2020

| **Variable** | **Depression (PHQ ≥ 10) *** | | | | **Anxiety (GAD ≥ 8) *** | | | |
| --- | --- | --- | --- | --- | --- | --- | --- | --- |
|  | **Model 1** | | **Model 2** | | **Model 1** | | **Model 2** | |
|  | **OR (95% CI)** | ***p*** | **OR (95% CI)** | ***p*** | **OR (95% CI)** | ***p*** | **OR (95% CI)** | ***p*** |
| Preventive behaviors^**^ |  |  |  |  |  |  |  |  |
| Low PB score (< 9.0) | 1.00 |  | 1.00 |  | 1.00 |  | 1.00 |  |
| High PB score (≥ 9.0) | 0.25 (0.21, 0.29) | <0.001 | 0.36 (0.30, 0.44) | <0.001 | 0.20 (0.17, 0.24) | <0.001 | 0.28 (0.23, 0.34) | <0.001 |
| Age groups |  |  |  |  |  |  |  |  |
| 18 – 39 | 1.00 |  | 1.00 |  | 1.00 |  | 1.00 |  |
| 40 – 59 | 1.57 (1.32, 1.88) | <0.001 | 1.64 (1.30, 2.06) | <0.001 | 2.04 (1.62, 2.57) | <0.001 | 1.72 (1.36, 2.19) | <0.001 |
| ≥ 60 | 5.25 (4.47, 6.16) | <0.001 | 4.77 (3.81, 5.96) | <0.001 | 9.93 (8.06, 12.2) | <0.001 | 6.07 (4.83, 7.62) | <0.001 |
| Gender |  |  |  |  |  |  |  |  |
| Women | 1.00 |  | 1.00 |  | 1.00 |  | 1.00 |  |
| Men | 0.99 (0.87, 1.13) | 0.906 | 0.93 (0.78, 1.11) | 0.429 | 1.12 (0.95, 1.31) | 0.182 | 0.87 (0.72, 1.05) | 0.148 |
| Occupation |  |  |  |  |  |  |  |  |
| Unemployed/ Dependents | 1.00 |  | 1.00 |  | 1.00 |  | 1.00 |  |
| Employed | 0.39 (0.32, 0.47) | <0.001 | 0.83 (0.64, 1.08) | 0.164 | 0.51 (0.41, 0.64) | <0.001 | 0.79 (0.61, 1.03) | 0.085 |
| Ability to pay for medication |  |  |  |  |  |  |  |  |
| Very or fairly difficult | 1.00 |  | 1.00 |  |  |  |  |  |
| Very or fairly easy | 0.37 (0.32, 0.43) | <0.001 | 0.57 (0.46, 0.71) | <0.001 |  |  |  |  |
| Social status |  |  |  |  |  |  |  |  |
| Low | 1.00 |  | 1.00 |  | 1.00 |  | 1.00 |  |
| Middle or high | 0.74 (0.63, 0.87) | <0.001 | 1.77 (1.40, 2.23) | <0.001 | 1.47 (1.19, 1.82) | <0.001 | 1.78 (1.40, 2.26) | <0.001 |
| Lockdown measure |  |  |  |  |  |  |  |  |
| No | 1.00 |  | 1.00 |  | 1.00 |  | 1.00 |  |
| Yes | 3.33 (2.91, 3.80) | <0.001 | 2.29 (1.89, 2.78) | <0.001 | 2.89 (2.42, 3.46) | <0.001 | 2.63 (2.16, 3.21) | <0.001 |

**Abbreviations** PHQ, patient health questionnaire; GAD, Generalized Anxiety Disorder; PB, preventive behaviors; OR, odd ratio; CI, confidence interval.

**^*^** Depression and Anxiety was analyzed on the sample of 4348 participants

**Table S11** Interactions of underlying health conditions with S-COVID-19-S on depression, and anxiety, Vietnam, 2020

| **Interaction** | **Depression (PHQ ≥ 10)** | | | | **Anxiety (GAD ≥ 8) *** | | | |
| --- | --- | --- | --- | --- | --- | --- | --- | --- |
|  | **Model 1** |  | **Model 2** |  | **Model 1** |  | **Model 2** |  |
|  | **OR (95% CI)** | ***p*** | **OR (95% CI)** | ***p*** | **OR (95% CI)** | ***p*** | **OR (95% CI)** | ***p*** |
| Non-UHC × Without S-COVID-19-S | 1.00 |  | 1.00 |  | 1.00 |  | 1.00 |  |
| Non-UHC × With S-COVID-19-S | 2.34 (1.96, 2.79) | <0.001 | 1.92 (1.59, 2.31) | <0.001 | 2.00 (1.56, 2.58) | <0.001 | 1.50 (1.15, 1.96) | 0.003 |
| UHC × Without S-COVID-19-S | 1.34 (0.98, 1.81) | 0.063 | 0.84 (0.61, 1.16) | 0.293 | 1.76 (1.19, 2.61) | 0.004 | 0.92 (0.61, 1.39) | 0.685 |
| UHC × With S-COVID-19-S | 2.77 (1.94, 3.94) | <0.001 | 2.62 (1.81, 3.79) | <0.001 | 2.18 (1.38, 3.45) | 0.001 | 3.12 (1.92, 5.07) | <0.001 |
| Age groups |  |  |  |  |  |  |  |  |
| 18 – 39 | 1.00 |  | 1.00 |  | 1.00 |  | 1.00 |  |
| 40 – 59 | 1.57 (1.32, 1.88) | <0.001 | 1.32 (1.09, 1.59) | 0.004 | 2.04 (1.62, 2.57) | <0.001 | 1.71 (1.34, 2.17) | <0.001 |
| ≥ 60 | 5.25 (4.47, 6.16) | <0.001 | 3.28 (2.74, 3.92) | <0.001 | 9.93 (8.06, 12.2) | <0.001 | 6.41 (5.09, 8.07) | <0.001 |
| Gender |  |  |  |  |  |  |  |  |
| Women | 1.00 |  | 1.00 |  | 1.00 |  | 1.00 |  |
| Men | 0.99 (0.87, 1.13) | 0.906 | 0.92 (0.79, 1.06) | 0.242 | 1.12 (0.95, 1.31) | 0.182 | 0.89 (0.73, 1.07) | 0.206 |
| Occupation |  |  |  |  |  |  |  |  |
| Unemployed/ Dependents | 1.00 |  | 1.00 |  | 1.00 |  | 1.00 |  |
| Employed | 0.39 (0.32, 0.47) | <0.001 | 0.69 (0.55, 0.86) | 0.001 | 0.51 (0.41, 0.64) | <0.001 | 0.69 (0.53, 0.91) | 0.008 |
| Ability to pay for treatments |  |  |  |  |  |  |  |  |
| Very or fairly difficult | 1.00 |  | 1.00 |  |  |  |  |  |
| Very or fairly easy | 0.37 (0.32, 0.43) | <0.001 | 0.64 (0.54, 0.76) | <0.001 |  |  |  |  |
| Social status |  |  |  |  |  |  |  |  |
| Low | 1.00 |  | 1.00 |  | 1.00 |  | 1.00 |  |
| Middle or high | 0.74 (0.63, 0.87) | <0.001 | 1.20 (0.99, 1.44) | 0.056 | 1.47 (1.19, 1.82) | <0.001 | 2.03 (1.59, 2.59) | <0.001 |
| Lockdown measure |  |  |  |  |  |  |  |  |
| No | 1.00 |  | 1.00 |  | 1.00 |  | 1.00 |  |
| Yes | 3.33 (2.91, 3.80) | <0.001 | 2.16 (1.85, 2.52) | <0.001 | 2.89 (2.42, 3.46) | <0.001 | 1.78 (1.46, 2.18) | <0.001 |

**Abbreviations** PHQ, patient health questionnaire; GAD, Generalized Anxiety Disorder; PB, preventive behaviors; OR, odd ratio; CI, confidence interval; UHC, underlying health conditions; S-COVID-19-S, suspected corona virus disease-2019 symptoms.

**^*^** Anxiety was analyzed on the sample of 4348 participants.

**Table S12** Interactions of underlying health conditions with drinking alcohol on anxiety, Vietnam, 2020

| **Interaction** | **Depression (PHQ ≥ 10)** | | | | **Anxiety (GAD ≥ 8) *** | | | |
| --- | --- | --- | --- | --- | --- | --- | --- | --- |
|  | **Model 1** |  | **Model 2** |  | **Model 1** |  | **Model 2** |  |
|  | **OR (95% CI)** | ***p*** | **OR (95% CI)** | ***p*** | **OR (95% CI)** | ***p*** | **OR (95% CI)** | ***p*** |
| Non-UHC × Never, stopped, or drink less |  |  |  |  | 1.00 |  | 1.00 |  |
| Non-UHC × Unchanged or drink more |  |  |  |  | 3.05 (2.21, 4.21) | <0.001 | 3.26 (2.28, 4.65) | <0.001 |
| UHC × Never, stopped, or drink less |  |  |  |  | 5.71 (4.77, 6.83) | <0.001 | 3.31 (2.71, 4.05) | <0.001 |
| UHC × Unchanged or drink more |  |  |  |  | 0.25 (0.12, 0.48) | <0.001 | 0.34 (0.16, 0.69) | 0.003 |
| Age groups |  |  |  |  |  |  |  |  |
| 18 – 39 |  |  |  |  | 1.00 |  | 1.00 |  |
| 40 – 59 |  |  |  |  | 2.04 (1.62, 2.57) | <0.001 | 1.66 (1.30, 2.11) | <0.001 |
| ≥ 60 |  |  |  |  | 9.93 (8.06, 12.2) | <0.001 | 6.07 (4.83, 7.63) | <0.001 |
| Gender |  |  |  |  |  |  |  |  |
| Women |  |  |  |  | 1.00 |  | 1.00 |  |
| Men |  |  |  |  | 1.12 (0.95, 1.31) | 0.182 | 0.77 (0.64, 0.94) | 0.008 |
| Occupation |  |  |  |  |  |  |  |  |
| Unemployed/ Dependents |  |  |  |  | 1.00 |  | 1.00 |  |
| Employed |  |  |  |  | 0.51 (0.41, 0.64) | <0.001 | 0.72 (0.55, 0.94) | 0.017 |
| Ability to pay for medication |  |  |  |  |  |  |  |  |
| Very or fairly difficult |  |  |  |  |  |  |  |  |
| Very or fairly easy |  |  |  |  |  |  |  |  |
| Social status |  |  |  |  |  |  |  |  |
| Low |  |  |  |  | 1.00 |  | 1.00 |  |
| Middle or high |  |  |  |  | 1.47 (1.19, 1.82) | <0.001 | 2.09 (1.64, 2.65) | <0.001 |
| Lockdown measure |  |  |  |  |  |  |  |  |
| No |  |  |  |  | 1.00 |  | 1.00 |  |
| Yes |  |  |  |  | 2.89 (2.42, 3.46) | <0.001 | 2.04 (1.67, 2.50) | <0.001 |

**Abbreviations** PHQ, patient health questionnaire; GAD, Generalized Anxiety Disorder; PB, preventive behaviors; OR, odd ratio; CI, confidence interval; UHC, underlying health conditions.

**^*^** Anxiety was analyzed on the sample of 4348 participants.

**Table S13** Interactions of underlying health conditions and physical activity on depression, and anxiety, Vietnam, 2020

| **Interaction** | **Depression (PHQ ≥ 10)** | | | | **Anxiety (GAD ≥ 8) *** | | | |
| --- | --- | --- | --- | --- | --- | --- | --- | --- |
|  | **Model 1** |  | **Model 2** |  | **Model 1** |  | **Model 2** |  |
|  | **OR (95% CI)** | ***p*** | **OR (95% CI)** | ***p*** | **OR (95% CI)** | ***p*** | **OR (95% CI)** | ***p*** |
| Non-UHC × Never, stopped, or exercise less | 1.00 |  | 1.00 |  | 1.00 |  | 1.00 |  |
| Non-UHC × Unchanged or exercise more | 0.58 (0.48, 0.69) | <0.001 | 0.77 (0.64, 0.94) | 0.010 | 0.68 (0.53, 0.86) | 0.002 | 0.75 (0.58, 0.97) | 0.027 |
| UHC × Never, stopped, or exercise less | 5.26 (4.34, 6.39) | <0.001 | 3.03 (2.45, 3.74) | <0.001 | 6.08 (4.96, 7.46) | <0.001 | 3.46 (2.76, 4.35) | <0.001 |
| UHC × Unchanged or exercise more | 0.36 (0.27, 0.48) | <0.001 | 0.41 (0.30, 0.56) | <0.001 | 0.34 (0.23, 0.50) | <0.001 | 0.42 (0.28, 0.64) | <0.001 |
| Age groups |  |  |  |  |  |  |  |  |
| 18 – 39 | 1.00 |  | 1.00 |  | 1.00 |  | 1.00 |  |
| 40 – 59 | 1.57 (1.32, 1.88) | <0.001 | 1.36 (1.12, 1.64) | 0.001 | 2.04 (1.62, 2.57) | <0.001 | 1.65 (1.29, 2.09) | <0.001 |
| ≥ 60 | 5.25 (4.47, 6.16) | <0.001 | 3.39 (2.84, 4.06) | <0.001 | 9.93 (8.06, 12.2) | <0.001 | 5.94 (4.72, 7.47) | <0.001 |
| Gender |  |  |  |  |  |  |  |  |
| Women | 1.00 |  | 1.00 |  | 1.00 |  | 1.00 |  |
| Men | 0.99 (0.87, 1.13) | 0.906 | 0.91 (0.79, 1.05) | 0.196 | 1.12 (0.95, 1.31) | 0.182 | 0.86 (0.71, 1.04) | 0.116 |
| Occupation |  |  |  |  |  |  |  |  |
| Unemployed/ Dependents | 1.00 |  | 1.00 |  | 1.00 |  | 1.00 |  |
| Employed | 0.39 (0.32, 0.47) | <0.001 | 0.71 (0.57, 0.89) | 0.003 | 0.51 (0.41, 0.64) | <0.001 | 0.71 (0.54, 0.93) | 0.012 |
| Ability to pay for medication |  |  |  |  |  |  |  |  |
| Very or fairly difficult | 1.00 |  | 1.00 |  |  |  |  |  |
| Very or fairly easy | 0.37 (0.32, 0.43) | <0.001 | 0.60 (0.51, 0.71) | <0.001 |  |  |  |  |
| Social status |  |  |  |  |  |  |  |  |
| Low | 1.00 |  | 1.00 |  | 1.00 |  | 1.00 |  |
| Middle or high | 0.74 (0.63, 0.87) | <0.001 | 1.22 (1.01, 1.47) | 0.035 | 1.47 (1.19, 1.82) | <0.001 | 2.03 (1.60, 2.59) | <0.001 |
| Lockdown measure |  |  |  |  |  |  |  |  |
| No | 1.00 |  | 1.00 |  | 1.00 |  | 1.00 |  |
| Yes | 3.33 (2.91, 3.80) | <0.001 | 1.89 (1.61, 2.22) | <0.001 | 2.89 (2.42, 3.46) | <0.001 | 1.81 (1.48, 2.21) | <0.001 |

**Abbreviations** PHQ, patient health questionnaire; GAD, Generalized Anxiety Disorder; PB, preventive behaviors; OR, odd ratio; CI, confidence interval; UHC, underlying health conditions.

**^*^** Anxiety was analyzed on the sample of 4348 participants

**Table S14** Interactions of underlying health conditions and eating behavior on depression, and anxiety, Vietnam, 2020

| **Interaction** | **Depression (PHQ ≥ 10)** | | | | **Anxiety (GAD ≥ 8) *** | | | |
| --- | --- | --- | --- | --- | --- | --- | --- | --- |
|  | **Model 1** |  | **Model 2** |  | **Model 1** |  | **Model 2** |  |
|  | **OR (95% CI)** | ***p*** | **OR (95% CI)** | ***p*** | **OR (95% CI)** | ***p*** | **OR (95% CI)** | ***p*** |
| Non-UHC × Less healthy | 1.00 |  | 1.00 |  | 1.00 |  | 1.00 |  |
| Non-UHC × Unchanged or healthier | 0.30 (0.21, 0.42) | <0.001 | 0.36 (0.25, 0.52) | <0.001 | 0.35 (0.23, 0.53) | <0.001 | 0.36 (0.23, 0.56) | <0.001 |
| UHC × Less healthy | 10.92 (6.79, 17.56) | <0.001 | 4.27 (2.57, 7.08) | <0.001 | 16.97 (9.78, 29.45) | <0.001 | 6.27 (3.46, 11.35) | <0.001 |
| UHC × Unchanged or healthier | 0.28 (0.17, 0.45) | <0.001 | 0.42 (0.25, 0.72) | 0.001 | 0.21 (0.12, 0.38) | <0.001 | 0.37 (0.20, 0.68) | 0.002 |
| Age groups |  |  |  |  |  |  |  |  |
| 18 – 39 | 1.00 |  | 1.00 |  | 1.00 |  | 1.00 |  |
| 40 – 59 | 1.57 (1.32, 1.88) | <0.001 | 1.35 (1.11, 1.63) | 0.002 | 2.04 (1.62, 2.57) | <0.001 | 1.64 (1.29, 2.09) | <0.001 |
| ≥ 60 | 5.25 (4.47, 6.16) | <0.001 | 3.19 (2.66, 3.82) | <0.001 | 9.93 (8.06, 12.2) | <0.001 | 5.67 (4.49, 7.17) | <0.001 |
| Gender |  |  |  |  |  |  |  |  |
| Women | 1.00 |  | 1.00 |  | 1.00 |  | 1.00 |  |
| Men | 0.99 (0.87, 1.13) | 0.906 | 0.90 (0.78, 1.04) | 0.167 | 1.12 (0.95, 1.31) | 0.182 | 0.86 (0.71, 1.04) | 0.113 |
| Occupation |  |  |  |  |  |  |  |  |
| Unemployed/ Dependents | 1.00 |  | 1.00 |  | 1.00 |  | 1.00 |  |
| Employed | 0.39 (0.32, 0.47) | <0.001 | 0.71 (0.57, 0.89) | 0.003 | 0.51 (0.41, 0.64) | <0.001 | 0.71 (0.54, 0.94) | 0.015 |
| Ability to pay for medication |  |  |  |  |  |  |  |  |
| Very or fairly difficult | 1.00 |  | 1.00 |  |  |  |  |  |
| Very or fairly easy | 0.37 (0.32, 0.43) | <0.001 | 0.59 (0.50, 0.70) | <0.001 |  |  |  |  |
| Social status |  |  |  |  |  |  |  |  |
| Low | 1.00 |  | 1.00 |  | 1.00 |  | 1.00 |  |
| Middle or high | 0.74 (0.63, 0.87) | <0.001 | 1.12 (0.94, 1.36) | 0.201 | 1.47 (1.19, 1.82) | <0.001 | 1.91 (1.50, 2.44) | <0.001 |
| Lockdown measure |  |  |  |  |  |  |  |  |
| No | 1.00 |  | 1.00 |  | 1.00 |  | 1.00 |  |
| Yes | 3.33 (2.91, 3.80) | <0.001 | 2.10 (1.81, 2.45) | <0.001 | 2.89 (2.42, 3.46) | <0.001 | 1.71 (1.40, 2.09) | <0.001 |

**Abbreviations** PHQ, patient health questionnaire; GAD, Generalized Anxiety Disorder; PB, preventive behaviors; OR, odd ratio; CI, confidence interval; UHC, underlying health conditions.

**^*^** Anxiety was analyzed on the sample of 4348 participants

**Table S15** Interactions of underlying health conditions and preventive behaviors on depression, and anxiety, Vietnam, 2020

| **Interaction** | **Depression (PHQ ≥ 10) *** | | | | **Anxiety (GAD ≥ 8) *** | | | |
| --- | --- | --- | --- | --- | --- | --- | --- | --- |
|  | **Model 1** |  | **Model 2** |  | **Model 1** |  | **Model 2** |  |
|  | **OR (95% CI)** | ***p*** | **OR (95% CI)** | ***p*** | **OR (95% CI)** | ***p*** | **OR (95% CI)** | ***p*** |
| Non-UHC × PB < 9.0 | 1.00 |  | 1.00 |  | 1.00 |  | 1.00 |  |
| Non-UHC × PB ≥ 9.0 | 0.49 (0.38, 0.62) | <0.001 | 0.57 (0.44, 0.73) | <0.001 | 0.48 (0.37, 0.61) | <0.001 | 0.55 (0.43, 0.71) | <0.001 |
| UHC × PB < 9.0 | 9.83 (7.75, 12.46) | <0.001 | 5.32 (4.11, 6.89) | <0.001 | 11.46 (9.02, 14.58) | <0.001 | 6.24 (4.80, 8.11) | <0.001 |
| UHC × PB ≥ 9.0 | 0.21 (0.15, 0.30) | <0.001 | 0.31 (0.21, 0.45) | <0.001 | 0.14 (0.09, 0.19) | <0.001 | 0.18 (0.12, 0.27) | <0.001 |
| Age groups |  |  |  |  |  |  |  |  |
| 18 – 39 | 1.00 |  | 1.00 |  | 1.00 |  | 1.00 |  |
| 40 – 59 | 1.57 (1.32, 1.88) | <0.001 | 1.31 (1.03, 1.66) | 0.029 | 2.04 (1.62, 2.57) | <0.001 | 1.37 (1.07, 1.77) | 0.013 |
| ≥ 60 | 5.25 (4.47, 6.16) | <0.001 | 3.02 (2.36, 3.85) | <0.001 | 9.93 (8.06, 12.2) | <0.001 | 3.83 (2.99, 4.92) | <0.001 |
| Gender |  |  |  |  |  |  |  |  |
| Women | 1.00 |  | 1.00 |  | 1.00 |  | 1.00 |  |
| Men | 0.99 (0.87, 1.13) | 0.906 | 0.91 (0.75, 1.10) | 0.333 | 1.12 (0.95, 1.31) | 0.182 | 0.85 (0.69, 1.03) | 0.107 |
| Occupation |  |  |  |  |  |  |  |  |
| Unemployed/ Dependents | 1.00 |  | 1.00 |  | 1.00 |  | 1.00 |  |
| Employed | 0.39 (0.32, 0.47) | <0.001 | 0.83 (0.63, 1.09) | 0.190 | 0.51 (0.41, 0.64) | <0.001 | 0.79 (0.59, 1.05) | 0.101 |
| Ability to pay for medication |  |  |  |  |  |  |  |  |
| Very or fairly difficult | 1.00 |  | 1.00 |  |  |  |  |  |
| Very or fairly easy | 0.37 (0.32, 0.43) | <0.001 | 0.61 (0.49, 0.77) | <0.001 |  |  |  |  |
| Social status |  |  |  |  |  |  |  |  |
| Low | 1.00 |  | 1.00 |  | 1.00 |  | 1.00 |  |
| Middle or high | 0.74 (0.63, 0.87) | <0.001 | 1.74 (1.36, 2.22) | <0.001 | 1.47 (1.19, 1.82) | <0.001 | 1.76 (1.36, 2.26) | <0.001 |
| Lockdown measure |  |  |  |  |  |  |  |  |
| No | 1.00 |  | 1.00 |  | 1.00 |  | 1.00 |  |
| Yes | 3.33 (2.91, 3.80) | <0.001 | 1.84 (1.50, 2.24) | <0.001 | 2.89 (2.42, 3.46) | <0.001 | 2.09 (1.69, 2.57) | <0.001 |

**Abbreviations** PHQ, patient health questionnaire; GAD, Generalized Anxiety Disorder; PB, preventive behaviors; PB, preventive behaviors; OR, odd ratio; CI, confidence interval; UHC, underlying health conditions.

**^*^** Depression and Anxiety was analyzed on the sample of 4348 participants

**Table S16**. Associations of underlying health conditions, suspected COVID-19 symptoms, health-related behavior changes, and preventive behaviors with depression, and anxiety using logistic regression models (further adjusted for the type of the administered questionnaire)

| **Variables** | **Depression (PHQ ≥10)** | | **Anxiety (GAD ≥8)** | |
| --- | --- | --- | --- | --- |
|  | **OR (95% CI)** | ***p**** | **OR (95% CI)** | ***p***** |
| Underlying health conditions |  |  |  |  |
| Non-UHC | 1.00 |  | 1.00 |  |
| UHC | 2.17 (1.86, 2.53) | <0.001 | 3.11 (2.55, 3.77) | <0.001 |
| S-COVID-19-S |  |  |  |  |
| No | 1.00 |  | 1.00 |  |
| Yes | 2.95 (2.54, 3.43) | <0.001 | 3.11 (2.56, 3.76) | <0.001 |
| Smoking tobacco |  |  |  |  |
| Never, stopped, or smoke less | 1.00 |  | 1.00 |  |
| Unchanged or smoke more | 1.07 (0.84, 1.38) | 0.573 | 1.02 (0.73, 1.42) | 0.924 |
| Drinking alcohol |  |  |  |  |
| Never, stopped, or drink less | 1.00 |  | 1.00 |  |
| Unchanged or drink more | 1.19 (0.97, 1.45) | 0.091 | 1.96 (1.43, 2.69) | <0.001 |
| Physical activity |  |  |  |  |
| Never, stopped, or exercise less | 1.00 |  | 1.00 |  |
| Unchanged or exercise more | 0.53 (0.45, 0.62) | <0.001 | 0.48 (0.39, 0.59) | <0.001 |
| Eating behavior |  |  |  |  |
| Less healthy | 1.00 |  | 1.00 |  |
| Unchanged or healthier | 0.22 (0.17, 0.28) | <0.001 | 0.19 (0.15, 0.25) | <0.001 |
| Preventive behaviors^**^ |  |  |  |  |
| Low PB score (< 9.0) | 1.00 |  | 1.00 |  |
| High PB score (≥ 9.0) | 0.36 (0.30, 0.44) | <0.001 | 0.29 (0.24, 0.35) | <0.001 |

**Abbreviations** PHQ, patient health questionnaire; GAD, Generalized Anxiety Disorder; UHC, underlying health conditions; S-COVID-19-S, suspected corona virus disease-2019 symptoms; PB, preventive behaviors; OR, odd ratio; CI, confidence interval.

* Adjusted for age, gender, occupation, ability to pay for treatments, lockdown measure, social status, and the type of the administered questionnaire.

** Adjusted for age, gender, occupation, lockdown measure, social status, and the type of the administered questionnaire.

**Table S17.** Interactions of underlying health conditions with suspected COVID-19 symptoms on depression, and anxiety (further adjusted for the type of the administered questionnaire).

| **Interaction** | **Depression (PHQ ≥10)** | | **Anxiety (GAD ≥8)** | |
| --- | --- | --- | --- | --- |
|  | **OR (95% CI)** | ***p**** | **OR (95% CI)** | ***p***** |
| Non-UHC × Without S-COVID-19-S | 1.00 |  | 1.00 |  |
| Non-UHC × With S-COVID-19-S | 1.99 (1.65, 2.41) | <0.001 | 1.57 (1.20, 2.05) | 0.001 |
| UHC × Without S-COVID-19-S | 0.87 (0.63, 1.20) | 0.399 | 0.95 (0.63, 1.44) | 0.812 |
| UHC × With S-COVID-19-S | 2.60 (1.79, 3.77) | <0.001 | 3.43 (2.11, 5.57) | <0.001 |

**Abbreviations** PHQ, patient health questionnaire; GAD, Generalized Anxiety Disorder; OR, odd ratio; CI, confidence interval; UHC, underlying health conditions; S-COVID-19-S, suspected corona virus disease-2019 symptoms.

* Adjusted for age, gender, occupation, ability to pay for treatments, lockdown measure, social status, and the type of the administered questionnaire.

** Adjusted for age, gender, occupation, lockdown measure, social status, and the type of the administered questionnaire.

**Table S18.** Interactions of underlying health conditions with health-related behavior changes on depression, and anxiety (further adjusted for the type of the administered questionnaire).

| **Interactions** | **Depression (PHQ ≥10)** | | **Anxiety (GAD ≥8)** | |
| --- | --- | --- | --- | --- |
|  | **OR (95% CI)** | ***p**** | **OR (95% CI)** | ***p***** |
| **Interaction of comorbidity with drinking alcohol** |  |  |  |  |
| Non-UHC × Never, stopped, or drink less |  |  | 1.00 |  |
| Non-UHC × Unchanged or drink more |  |  | 3.22 (2.26, 4.61) | <0.001 |
| UHC × Never, stopped, or drink less |  |  | 3.59 (2.92, 4.42) | <0.001 |
| UHC × Unchanged or drink more |  |  | 0.32 (0.16, 0.67) | 0.002 |
| **Interaction of comorbidity with physical activity** |  |  |  |  |
| Non-UHC × Never, stopped, or exercise less | 1.00 |  | 1.00 |  |
| Non-UHC × Unchanged or exercise more | 0.81 (0.67. 0.99) | 0.045 | 0.75 (0.58, 0.97) | 0.026 |
| UHC × Never, stopped, or exercise less | 3.14 (2.53, 3.88) | <0.001 | 3.74 (2.96, 4.73) | <0.001 |
| UHC × Unchanged or exercise more | 0.40 (0.29, 0.55) | <0.001 | 0.42 (0.28, 0.64) | <0.001 |
| **Interaction of comorbidity with eating behavior** |  |  |  |  |
| Non-UHC × Less healthy | 1.00 |  | 1.00 |  |
| Non-UHC × Unchanged or healthier | 0.37 (0.25, 0.53) | <0.001 | 0.35 (0.23, 0.55) | <0.001 |
| UHC × Less healthy | 4.45 (2.68, 7.38) | <0.001 | 6.65 (3.67, 12.06) | <0.001 |
| UHC × Unchanged or healthier | 0.42 (0.25, 0.71) | 0.001 | 0.38 (0.20, 0.71) | 0.002 |

**Abbreviations** PHQ, patient health questionnaire; GAD, Generalized Anxiety Disorder; OR, odd ratio; CI, confidence interval; UHC, underlying health conditions.

* Adjusted for age, gender, occupation, ability to pay for treatments, lockdown measure, social status, and the type of the administered questionnaire.

** Adjusted for age, gender, occupation, lockdown measure, social status, and the type of the administered questionnaire.

**Table S19.** Interactions of underlying health conditions with preventive behaviors on depression, and anxiety (further adjusted for the type of the administered questionnaire).

| **Interaction** | **Depression (PHQ ≥10)** | | **Anxiety (GAD ≥8)** | |
| --- | --- | --- | --- | --- |
|  | **OR (95% CI)** | ***p**** | **OR (95% CI)** | ***p***** |
| Non-UHC × PB score < 9.0 | 1.00 |  | 1.00 |  |
| Non-UHC × PB score ≥ 9.0 | 0.57 (0.44, 0.73) | <0.001 | 0.55 (0.43, 0.71) | <0.001 |
| UHC × PB score < 9.0 | 5.32 (4.11, 6.89) | <0.001 | 6.27 (4.82, 8.15) | <0.001 |
| UHC × PB score ≥ 9.0 | 0.31 (0.21, 0.45) | <0.001 | 0.19 (0.13, 0.28) | <0.001 |

**Abbreviations** PHQ, patient health questionnaire; GAD, Generalized Anxiety Disorder; OR, odd ratio; CI, confidence interval; UHC, underlying health conditions; PB, preventive behaviors.

* Adjusted for age, gender, occupation, ability to pay for treatments, lockdown measure, social status, and the type of the administered questionnaire.

** Adjusted for age, gender, occupation, lockdown measure, social status, and the type of the administered questionnaire.
